# Efflux Pump-Binding 4(3-Aminocyclobutyl)Pyrimidin-2-Amines Are Colloidal Aggregators

**DOI:** 10.3390/biom13061000

**Published:** 2023-06-16

**Authors:** Tania Szal, Shweta Singh Chauhan, Philipp Lewe, Fatima-Zahra Rachad, Marina Madre, Laura Paunina, Susanne Witt, Ramakrishnan Parthasarathi, Björn Windshügel

**Affiliations:** 1 Fraunhofer Institute for Translational Medicine and Pharmacology ITMP, Discovery Research ScreeningPort, 22525 Hamburg, Germany; tania.szal@itmp.fraunhofer.de (T.S.); fatima-zahra.rachad@itmp.fraunhofer.de (F.-Z.R.); 2 School of Science, Constructor University, 28759 Bremen, Germany; 3 Computational Toxicology Facility, Toxicoinformatics & Industrial Research CSIR-Indian Institute of Toxicology Research, Vishvigyan Bhavan, 31, Mahatma Gandhi Marg, Lucknow 226001, Uttar Pradesh, India; shwetasingh1chauhan@gmail.com (S.S.C.); partha.ram@iitr.res.in (R.P.); 4 Academy of Scientific and Innovative Research (AcSIR), Ghaziabad 201002, Uttar Pradesh, India; 5 Centre for Structural Systems Biology (CSSB), University Medical Center Hamburg-Eppendorf (UKE), 22607 Hamburg, Germany; philipp.lewe@cssb-hamburg.de (P.L.); susanne.witt@cssb-hamburg.de (S.W.); 6 Latvian Institute of Organic Synthesis, LV-1006 Riga, Latvia; madre@osi.lv (M.M.); laurapaunina@gmail.com (L.P.)

**Keywords:** efflux pumps, AcrA, efflux pump inhibitor, *E. coli*, virtual screening, antimicrobial studies, microscale thermophoresis, dynamic light scattering, nano-differential scanning fluorimetry, colloidal aggregators, assay interference

## Abstract

Efflux pumps are a relevant factor in antimicrobial resistance. In *E. coli*, the tripartite efflux pump AcrAB-TolC removes a chemically diverse set of antibiotics from the bacterium. Therefore, small molecules interfering with efflux pump function are considered adjuvants for improving antimicrobial therapies. Several compounds targeting the periplasmic adapter protein AcrA and the efflux pump AcrB have been identified to act synergistically with different antibiotics. Among those, several 4(3-aminocyclobutyl)pyrimidin-2-amines have been shown to bind to both proteins. In this study, we intended to identify analogs of these substances with improved binding affinity to AcrA using virtual screening followed by experimental validation. While we succeeded in identifying several compounds showing a synergistic effect with erythromycin on *E. coli*, biophysical studies suggested that 4(3-aminocyclobutyl)pyrimidin-2-amines form colloidal aggregates that do not bind specifically to AcrA. Therefore, these substances are not suited for further development. Our study emphasizes the importance of implementing additional control experiments to identify aggregators among bioactive compounds.

## 1. Introduction

The growing resistance of bacteria against antibiotics is a global threat to public health [1]. According to a recent study, infections with drug-resistant bacteria were directly responsible for 1.27 million deaths worldwide in 2019 [2]. A few years ago, the WHO published a priority pathogens list for research and development of new antibiotics that includes bacteria for which new antibiotics are urgently needed, including third-generation cephalosporin-resistant *E. coli* [3]. In particular, infections with bacteria of the ESKAPE panel, all belonging to the critical and high category of the WHO priority pathogens list, pose a tremendous threat as this group of pathogens shows growing antimicrobial resistance and virulence [4].

Most pathogens of concern are represented by Gram-negative bacteria that have developed diverse mechanisms for reducing antibiotic permeation and accumulation [5]. The outer membrane limits the accessibility of antibiotics to their intracellular targets, and through alteration of the expression of porin proteins, the susceptibility of bacteria to various antibiotics can be further limited [5]. In addition, efflux pumps extrude antibiotics from the bacterium, which results in sub-inhibitory drug concentrations. To date, six families of efflux pumps have been identified, of which members of the resistance-nodulation-cell division (RND) superfamily are of particular concern with respect to multidrug resistance among clinically relevant Gram-negative bacteria [6,7].

An attractive approach to reviving the efficacy of known antibiotics is the use of antibiotic adjuvants [8]. These substances possess very limited or no intrinsic antimicrobial activity but instead act by β-lactamase inhibition, membrane permeabilization, or efflux inhibition. In particular, efflux inhibition is of interest, as efflux pumps have a broad substrate spectrum, and compounds interfering with antibiotics efflux improve the efficacy of these drugs [9].

The best-characterized efflux pump is the AcrAB-TolC complex in *E. coli*, which is composed of the outer membrane factor TolC, the periplasmic adapter protein AcrA, and the RND-type efflux pump AcrB embedded in the inner membrane [10]. Assembled in a 3:6:3 stoichiometry, the complex spans the inner and outer membrane and removes substrates from the periplasmic space as well as the cytoplasm. In recent years, various studies have reported the identification of compounds targeting the AcrAB-TolC efflux pump in *E. coli*. For AcrB, a diverse set of small-molecule inhibitors have been discovered that shift the minimum inhibitory concentration (MIC) of chemically distinct antibiotics such as novobiocin or erythromycin, for example PAβN, NMP, and BDM88855.HCl [11,12]. In contrast, only a few compounds targeting AcrA have been reported, including the polybasic terephthalic acid derivative NSC 60339, which shifts the MICs of novobiocin or erythromycin [13,14]. In addition, several 4(3-aminocyclobutyl)pyrimidin-2-amines have been described to bind to AcrA with equilibrium dissociation constants of around 20 µM [15]. However, the compounds did not prevent the efflux of the fluorescent stain Hoechst 33342 from the cell, which is a standard method for assessing compound-mediated blockage of efflux pumps. The authors still believed the substances to be efflux pump inhibitors (EPIs) due to observed binding to AcrA and AcrB as measured using surface plasmon resonance (SPR) spectroscopy [15].

Based on 4(3-aminocyclobutyl)pyrimidin-2-amines, we intended to identify analogs with improved binding affinity for AcrA. For this purpose, we conducted a virtual screen, followed by experimental validation and characterization of selected substances. While we succeeded in identifying compounds with improved synergistic activity in combination with erythromycin, our binding studies indicated that compounds possessing this chemical scaffold bind to AcrA in a nonspecific manner. Further investigations suggested that the compounds are colloidal aggregators that promiscuously interact with proteins and disturb a range of different assays.

## 2. Materials and Methods

### 2.1. AcrA Protein Modeling

As no full-length structure of AcrA is available, a homology modeling approach was chosen. To identify a suited modeling template, a protein BLAST search was conducted using the sequence of *E. coli* AcrA, downloaded from UniProt. Eleven sequences with their specific PDB IDs were identified as showing similarity with AcrA. Based on structure analysis of the putative modeling templates, a homology model of AcrA was generated from a partially complete X-ray crystal structure of AcrA (PDB ID 2F1M) and the cryo-EM structure (PDB ID 5NG5). Upon alignment of the query sequence with the selected templates, altogether, 100 homology models were generated using MODELLER 9.21 [16]. Based on the DOPE and the GA341 scores, one homology model was chosen for docking studies.

### 2.2. Virtual Screening

A similarity search within the ZINC database [17] was carried out to identify and download commercially available 4(3-aminocyclobutyl)pyrimidin-2-amine analogs. The AcrA protein model was prepared (addition of hydrogens) using AutoDockTools [18]. All selected compounds were docked into the AcrA protein model using AutoDock Vina [19].

### 2.3. Antimicrobial Potentiation Assay

Antimicrobial checkerboard assays in a 96-well plate format were carried out according to a previously described method [20]. Equal amounts (66 µL each) of *E. coli* BW25113 bacterial suspension (2.5 × 10^5^ CFU; DSMZ, Braunschweig, Germany) and erythromycin and compound solutions, diluted into Luria Bertani (Miller) Broth (LB Broth; Carl Roth GmbH + Co. KG, Karlsruhe, Germany), were added to the plate. Test compounds were obtained from commercial suppliers (MolPort, Riga, Latvia; Ambinter, Orléans, France). Compound 24123034 was synthesized (see Supporting Information). The intrinsic MIC of each test compound and erythromycin (Sigma Aldrich, Merck KGaA, Darmstadt, Germany) against *E. coli* BW25113 was determined on the same plate. Compound 36287038 [15] was used as a positive control in potentiation assays at a fixed concentration of 25 µM. Compounds were tested at concentrations ranging from 400 to 12.5 µM (100 to 25 µM for compounds 3, 4, 27, 39, 44, 47, 48), while erythromycin was applied in concentrations ranging from 2 to 256 µg/mL. The highest DMSO concentration was 0.9%. The plates were shaken overnight at 37 °C and 150 rpm with 96-well plate sandwich lids to prevent evaporation (CR1296, EnzyScreen BV, Heemstede, Netherlands). The optical density at 600 nm (OD_600nm_) was measured the next day using a Fusion α-FP HT Microplate Reader (PerkinElmer, Rodgau, Germany). Three independent experiments were carried out. For analysis, the DMSO control was subtracted from each well. The “no growth” limit was defined as the mean value of the antibiotic control (1536 µg/mL) plus three standard deviations. MPC_4_ values were assessed as the lowest compound concentration that is specified as “no growth” at one-fourth of the determined MIC of erythromycin (128 to 256 µg/mL) and presented as the mean with standard deviation.

Antimicrobial potentiation assays for compound 24123034 were likewise performed at 25 and 50 µM compound concentrations. The OD_600nm_ was measured using an EnSpire^®^ multimode plate reader (PerkinElmer, Rodgau, Germany).

In addition, the synergistically acting compounds (24123034, 36287038, and 45) were investigated for the potentiation of erythromycin in efflux-deficient strains *E. coli* C43 (DE3) ΔAcrAB, kindly provided by Klaas Martinus Pos from Goethe University Frankfurt (Germany), and *E. coli* TolC (DSMZ104619, DSMZ, Braunschweig, Germany) at 25 and 50 µM concentrations and in the presence of a serial dilution of erythromycin (MIC: 2 µg/mL).

### 2.4. Cell Viability Studies

Eukaryotic cytotoxicity was determined by measuring intracellular ATP concentrations after lysis using a CellTiter-Glo^®^ assay (Promega GmbH, Walldorf, Germany). A549, HEK293, and HepG2 (CLS Cell Lines Service GmbH, Eppelheim, Germany) were grown in Dulbecco’s Modified Eagle’s Medium (DMEM) (Gibco™, Thermo Fisher Scientific, Life Technologies GmbH, Darmstadt, Germany) supplemented with 10% FBS (Capricorn Scientific GmbH, Ebsdorfergrund, Germany), 2 mM L-glutamine (Capricorn Scientific GmbH, Ebsdorfergrund, Germany) and 100 U/mL penicillin combined with 0.1 mg/mL streptomycin (Thermo Fisher Scientific, Life Technologies GmbH, Darmstadt, Germany). A total of 2000 cells/well were plated for HepG2 and HEK293 and 1000 cells/well for A549 in 20 µL of medium in white solid 384-well CellStar plates (Greiner Bio-One GmbH, Frickenhausen, Germany). Plates were incubated for 24 h at 37 °C in a 5% CO_2_ atmosphere. Test compounds were assessed in a dose–response format (7-point 1:3 dilution series, starting concentration 40 µM for compounds 29, 35, and 45; 100 µM for compound 38). A total of 20 nL (compound 38) or 40 nL (compounds 29, 35, and 45) of each dilution was added to the plates using an Echo 550R Acoustic Liquid Handler (Labcyte Inc., San José, CA, USA). The final DMSO concentration was 0.1% or 0.2%. Assay plates were incubated for 48 h at 37 °C in a 5% CO_2_ atmosphere. After equilibration to room temperature for 30 min, 20 µL of the CellTiter-Glo^®^ reagent was added to each well, and the plate was shaken at 600 rpm for three minutes. The assay plates were centrifuged for 1 min at 1000 × *g* and incubated for 10 min in the dark at room temperature before luminescence was measured using an EnVision plate reader (PerkinElmer, Rodgau, Germany). The signals measured at each concentration were normalized to the mean of the signals of the growth control wells (0.1% or 0.2% *v*/*v* DMSO, 100% viability) and plotted against the concentrations of the compound using GraphPad Prism 9.2.0 (GraphPad Software, San Diego, CA, USA). Curves were fitted applying nonlinear regression curve using a four-parameter sigmoidal curve to assess CC_50_. The assay was repeated three times, with each concentration tested in at least triplicate. The specified CC_50_-values are the means of the replicates.

### 2.5. Protein Expression and Purification

The gene encoding AcrA without its signal peptide (amino acids 26 to 397) was amplified from a K12-derived *E. coli* strain and cloned into the NdeI and XhoI sites of pET21a. The integrity of the generated expression plasmid was confirmed through sequencing. For expression, *E. coli* BL21(DE3) cells were transformed with the AcrA pET21a expression plasmid and grown in LB media in the presence of the appropriate selection marker at 37 °C and 180 rpm to an OD_600nm_ of 0.6 and subsequently induced with 1 mM IPTG. Growth was continued for 4 h at 25 °C and 180 rpm. After cell harvest, the pellets were flash-frozen and stored at deep temperature until further use. For cell lysis, the cells were resuspended in 4 mL per g pellet in lysis buffer (20 mM Tris/HCl pH 7.5, 150 mM NaCl, 2 mM MgCl_2_, cOmplete™, Mini, EDTA-free Protease Inhibitor Cocktail (Sigma Aldrich, Merck KGaA, Taufkirchen, Germany), spatula DNAseI (AppliChem GmbH, Darmstadt, Germany)), and lysed using a sonotrode 106 Bandelin Sonicator (BANDELIN electronic GmbH & Co. KG, Berlin, Germany). The soluble fraction was cleared by means of centrifugation for 30 min at 30,000× *g* and 4 °C and applied to an appropriate His-trap column using an ÄKTA pure™ 25L (Cytiva, Freiburg, Germany) according to the manufacturer’s manual. The affinity chromatography purification step was followed by size exclusion chromatography using an S200 PG 16/600 column (Cytiva, Freiburg, Germany). Fractions containing dimeric AcrA_His_ were pooled, concentrated, aliquoted, and flash-frozen at −80 °C until further use.

For AcrB protein expression, *E.coli* C43 (DE3) ΔAcrAB cells were transformed with the plasmid pET24_acrB-His [21] and grown in TB media at 37 °C and 150 rpm to an OD_600nm_ of 0.8 and subsequently induced with 0.5 mM IPTG. Growth continued for 16 h at 20 °C and 130 rpm. After cell harvest, the pellets were flash-frozen at −80 °C and stored until further use. For cell lysis, the cells were resuspended in 4 mL per g pellet with lysis buffer (phosphate-buffered saline (PBS) pH 8, 500 mM NaCl, 2 mg DNase I (AppliChem GmbH, Darmstadt, Germany), cOmplete™, Mini EDTA-free Protease Inhibitor Cocktail (Sigma Aldrich, Merck KGaA, Taufkirchen, Germany)) and lysed using an LM 10 Microfluidizer. After cell lysis, the cell lysate was centrifuged at 5000× *g* for 20 min at 4 °C. The supernatant was centrifuged at 10,000× *g* for 30 min and subsequently at 137,000× *g* for one hour. The supernatant was discarded without destroying the pellet. For the membrane wash, the pellet was resuspended with lysis buffer and centrifuged at 137,000× *g* for one hour. The pellet was resuspended with lysis buffer, aliquoted into Falcon tubes, and stored at −80 °C. For solubilization, 1% DDM was used, and the soluble fraction was cleared by means of centrifugation for 30 min at 100,000× *g* (4 °C) and applied to an His-trap HP 5 mL column (Cytiva, Freiburg, Germany) using an ÄKTA pure™ 25L system according to the manufacturer’s manual. The affinity chromatography purification step was followed by a size exclusion chromatography step using an S200 16/600 PG column with PBS buffer (pH 7.5, 0.03% DDM). Fractions were pooled, concentrated, aliquoted, and flash-frozen at deep temperatures until further use.

Aliquots of TEV_His_ and _GST_3C were kindly provided by the Protein Production Core Facility at CSSB Hamburg. The expression cell line and the plasmid for the AcrB production were kindly provided by Klaas Martinus Pos from Goethe University Frankfurt (Frankfurt am Main, Germany).

### 2.6. Microscale Thermophoresis (MST)

Measurements were carried out in triplicate on a Monolith NT.115 (NanoTemper Technologies GmbH, Munich, Germany) at the Sample Preparation and Characterisation Facility at the Centre for Structural Systems Biology (CSSB)/EMBL Hamburg. The LED power was set to 1% and the IR laser power to 40%. Laser on- and off-times were 3 and 20 s while the temperature was adjusted to 22 °C. AcrA_His_ measurements were conducted in 20 mM HEPES/NaOH pH 8.0, 150 mM NaCl, 0.05% Tween^®^ 20. Measurements with TEVp_His_, AcrB_His_, and _GST_3C were conducted in PBS (pH 7.4) with 0.03% DDM (Carl Roth GmbH & Co KG, Karlsruhe, Germany). The concentration of AcrA_His_ and the control protein TEVp_His_ was 5 µM, while AcrB_His_ and _GST_3C were tested at 1.25 µM and 4 µM, respectively. Test compounds were titrated in a three- or five-fold dilution series in the respective buffer with 2.5% DMSO (starting concentration 8 mM). The highest final DMSO concentration was 2% and proved to be tolerated by AcrA_His_ without denaturation (Figure S1). All dilutions were incubated at room temperature for 15 min to reach equilibrium and measured in premium coated capillaries (NanoTemper Technologies GmbH, Munich, Germany) to avoid sticking of the proteins to the surface. For the experiments with AcrA_His_ and Pluronic^®^ F-127 (Sigma Aldrich, Merck KGaA, Taufkirchen, Germany), samples were prepared as before, except that Pluronic^®^ F-127 was added to a final concentration of 0.08%.

Data points that showed irregular capillary shape or differed by more than 20% in the initial fluorescence were removed (AcrA_His_ and control proteins) and the fluorescence traces were fitted. For the fitting of the AcrB_His_ signals, no data points were removed, and the initial fluorescence was used. The analysis was conducted with ThermoAffinity (https://spc.embl-hamburg.de; accessed on 21 November 2022) [22] using a one-site fitting. The confidence interval (marginal asymmetric confidence interval at a 95% confidence level) was estimated according to a previously described method [23]. Compounds with curves that could not yield a 95% confidence interval were interpreted as not binding, as well as compounds that resulted in curves with a signal-to-noise ratio (S/N) below five. The S/N ratio was calculated using the formula

SN=RASresiduals

$$\mathrm{RA}=C_{\mathrm{Prot}\;}\times \;\left(\mathrm{RF}1-\mathrm{RF}2\right)$$

RA: response amplitude

S_residuals_: standard deviation of the residuals

C_Prot_: protein concentration in measurement (µM) 

RF1: signal of 1 µM protein alone

RF2: signal of the protein–ligand complex

### 2.7. Dynamic Light Scattering (DLS)

Samples were prepared as described for the MST measurements. Three concentrations were selected (890 µM, 297 µM, and 33 µM) for the active compounds (for inactive compounds: 890 µM) and added to 5 µM AcrA_His_. The autocorrelation curves were measured with a DynaPro Plate Reader III (Wyatt Technology Europe GmbH, Dernbach, Germany) with an automatic adjustment of the laser power at 817 nm with a detector angle of 150°. All measurements were performed in a 384-well plate in quadruplicate (25 μL sample/well) at 20 °C. The plate was scanned three times, and every well was read 16 times for 1 s. The scans of each well and the replicates were averaged. The final autocorrelation curves were analyzed and plotted with the Raynals online tool (https://spc.embl-hamburg.de; accessed on 28 October 2022) [24] using the viscosity and refractive index of water for fitting. The regularization parameter was set to 0.0025.

### 2.8. Nano-Differential Scanning Fluorimetry (nDSF)

Samples were prepared as described for the DLS measurements. The compounds were tested at a 2 mM concentration. After incubation, the sample was loaded into Prometheus High Sensitivity Capillaries (NanoTemper Technologies GmbH, Munich, Germany). Samples were heated at 1 °C per minute. The temperature range (40 to 65 °C) was adapted to the melting temperature of AcrA_His_ (T_M_ = 50 °C). Concurrent turbidity measurements utilizing back-reflection technology were used to control the solubility of the compounds (Figure S2). All measurements were repeated three times. For melting temperature estimation, “Equilibrium Two State” fitting of the MoltenProt online tool (https://spc.embl-hamburg.de; accessed on 28 October 2022) [25] was used. 

## 3. Results

### 3.1. Identification and Validation of 4(3-Aminocyclobutyl)Pyrimidin-2-Amine Analogs

**Figure 1:**
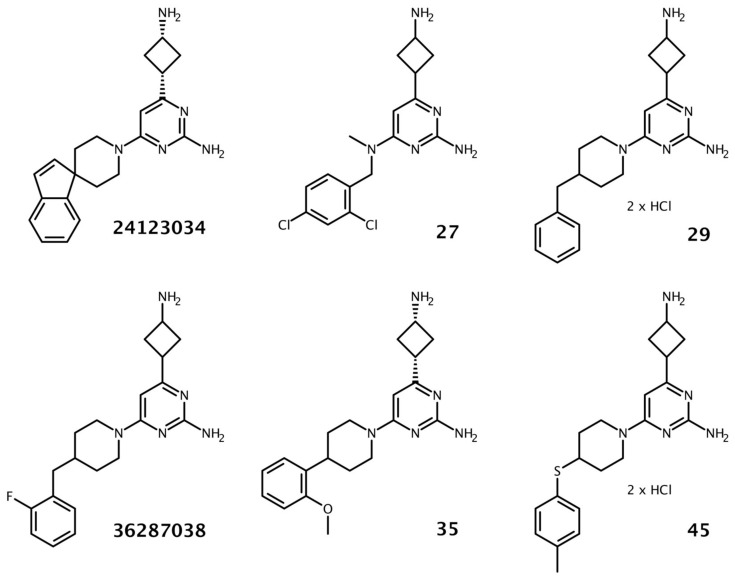
Two-dimensional structures of the previously reported 4(3-aminocyclobutyl)pyrimidin-2-amines 36287038 and 24123034 and selected analogs identified through the virtual screening campaign.

A structure-based approach was applied to identify novel analogs of the previously reported AcrA-binding compounds containing a 4(3-aminocyclobutyl)pyrimidin-2-amine scaffold. Initially, 3D structures of all compounds possessing this scaffold were retrieved from the ZINC database using a similarity search. A full-length structural model of AcrA was employed to dock all selected compounds into AcrA using AutoDock Vina. Based on the docking scores and visual inspection of the binding modes at the previously described binding sites on AcrA [15], 48 compounds were selected for experimental validation. Despite their structural similarity, the binding modes showed deviation from each other (Figure 1). 

Altogether, 48 compounds were available from chemical vendors, as well as the previously reported AcrA-binding compounds 24123034 and 36287038. The core structure of the majority of the substances was composed of two rings (a pyrimidine substituted with a cyclobutane) that mainly differed in the pyrimidine substitutions. Initially, a checkerboard assay was carried out to investigate any synergistic effect of the compounds in combination with erythromycin. A compound was defined as synergistically acting if it reduced the MIC (minimal inhibitory concentration) of erythromycin by at least a factor of four. The concentration that shifted the MIC of erythromycin (128 to 256 µg/mL) four-fold was considered the minimum potentiating concentration (MPC_4_) and was utilized to compare the compounds and to guide the selection for further characterization. Furthermore, the intrinsic antimicrobial activity of the compounds was assessed. Most compounds did not indicate any intrinsic antimicrobial activity up to the highest tested concentrations (either 100 or 400 µM), except compounds 36287038, 29, 30, 38, and 45 (200 to 400 µM). 

In combination with erythromycin, we observed a MPC_4_ of 20.8 µM for the positive control 36287038 and an MPC_4_ of 25 µM for 24123034 (*E. coli* BW25113), which is similar to the reported MPC_4_ obtained by using hyperporinated *E. coli* (25 and 12.5 µM) [15]. Compound 36287038 showed a low intrinsic antibacterial potential (MIC) that was about 20 times higher than the MPC_4_ (400 µM; Table S1), while compound 24123034 did not show any intrinsic antibacterial activity up to 50 µM. Three compounds (27, 29, 35) demonstrated a similar synergistic effect (MPC_4_ = 25 µM) compared to 36287038, while one compound (45) showed an MPC_4_ of 12.5 µM.

Next, we analyzed the structural differences between the compounds (Figure S3) and the associated changes in the synergistic effect with erythromycin (Tables S2–S6). Compounds containing a hydroxyl group instead of the amine substituent at the cyclobutane moiety showed no activity (1 to 10). Similarly, modifications of the amine moiety in the second position at the pyrimidine ring (11 and 12) resulted in a loss of synergism. Replacement of the piperidine ring (30) with piperazine (13) lowered the synergistic activity. Modification of the ring conformation by changing the piperazine to a piperazinone ring (18–22) resulted in a lower synergistic effect and thus a higher MPC_4_. Complete removal of the piperazine ring (27) proved to be tolerated for the synergistic activity (MPC_4_ = 25 µM). However, the replacement of the piperazine ring with a five- or seven-membered ring led to a loss of synergistic activity with erythromycin (23–26). Likewise, changing the aromatic substituent of the piperidine ring to a heteroaromatic ring (31) or non-cyclic moieties (32–34) resulted in a loss of synergism. The presence of substituents at the terminal phenyl ring improved the MPC_4_ (14) compared to an unsubstituted ring (13). The position of the substituents affected the synergistic activity. A meta-substituted compound (39) showed a slightly higher MPC_4_ compared to a para- (37) or ortho-substituted compound (38). Increasing the rigidity by introducing a carbonyl linker between the piperidine and the aromatic ring (46) resulted in a loss of synergistic activity. The most potent compound (45), having an MPC_4_ of 12.5 µM, contained a thioether linker. Lastly, the comparison of compounds 19 (MPC_4_ = 100 µM) and 21 (MPC_4_ = 133 µM), as well as 29 (MPC_4_ = 25 µM) and 30 (MPC_4_ = 50 µM), showed that the salt forms (hydrochlorides) of the molecules increased their synergistic activity, possibly due to the enhancement of solubility.

Previously, compounds 24123034 and 36287038 have been reported to possess no synergistic effect using a TolC-deficient strain [15]. Strikingly, in this study, both substances as well as compound 45 acted synergistically with erythromycin (MPC4 < 25 µM; Table S7) in an AcrA- and AcrB-deficient strain (*E. coli* C43 (DE3) ΔAcrAB) and a TolC-deficient strain (*E. coli* TolC). This indicates that the synergistic effect measured in the wildtype strain is not AcrA- or AcrB-dependent.

### 3.2. Cytotoxic Potential of 4(3-Aminocyclobutyl)Pyrimidin-2-Amine Analogs

In a further step, we assessed the cytotoxic potential of the three most potent compounds (29, 35, 45) and a less active substance (38) on three human cell lines using a cell viability assay. All compounds demonstrated a pronounced reduction in cell viability at low micromolar concentrations (Table S8). The CC_50_ (concentration of test compounds required to reduce cell viability by 50%) using liver carcinoma cells (HepG2), lung adenocarcinoma cells (A549), or immortalized embryonic kidney cells (HEK293) for compounds 38 and 45 ranged from 7.3 to 14.7 µM. Compounds 29 and 35 emerged as slightly less cytotoxic, with CC_50_ values ranging from 17.2 to 29.2 µM.

### 3.3. Protein Binding of Selected Compounds

**Figure 2:**
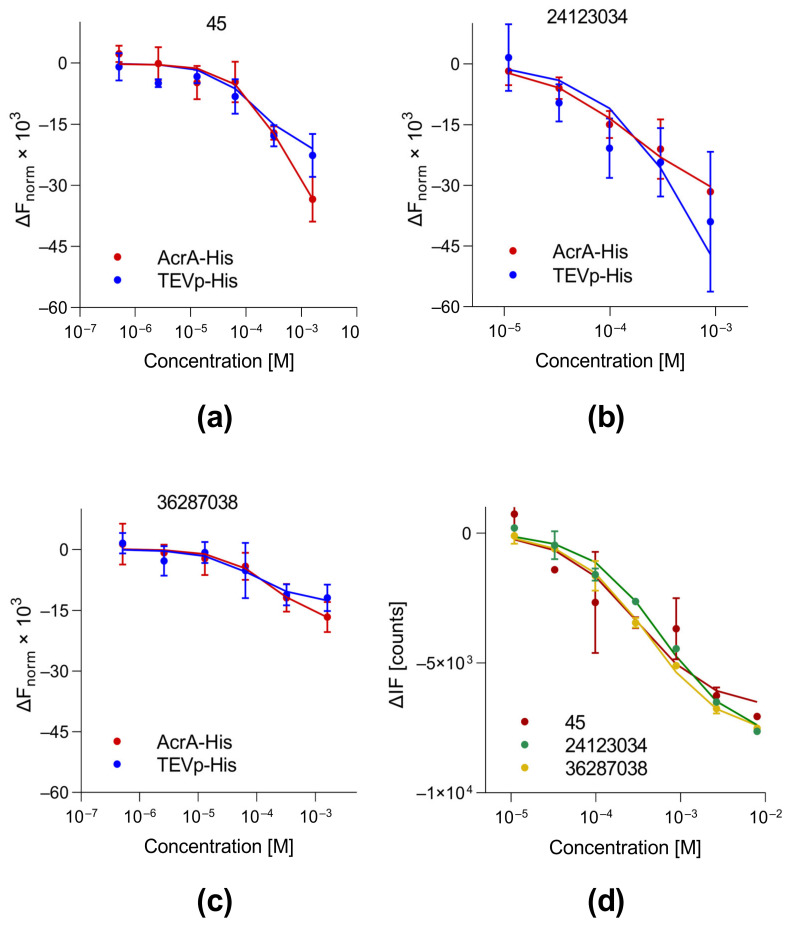
(**a–c**) Dose–response curves obtained through MST using His-tagged AcrA (red) or His-tagged TEVp (blue) in the presence of compounds 45, 24123034, and 36287038. (**d**) Binding curves obtained through MST (initial fluorescence) using His-tagged AcrB in the presence of compounds 45 (red), 24123034 (green), and 36287038 (yellow). (**a**–**d**) To facilitate comparison of binding data obtained for the different proteins, the baseline was subtracted from the curve and multiplied by 1000 for (**a**–**c**). Each data point is presented as mean ± SD (*n* = 3). Data points were derived from Figures S5–S13.

Next, we evaluated the binding affinity of compound 45 to His-tagged AcrA and AcrB using MST. In the case of AcrB_His_, quenching of the initial fluorescence (IF) of the protein occurred and was used to obtain the equilibrium dissociation constant (K_D_). Our measurements revealed that compound 45 interacts with both AcrA_His_ (K_D_ = 485 µM) and AcrB_His_ (K_D_ = 301 µM). For comparison, the already-published 4(3-aminocyclobutyl)pyrimidin-2-amine analogs 36287038 and 24123034 were tested for AcrA_His_ and AcrB_His_ binding. The values obtained for AcrA (24123034 = 164 µM; 36287038 = 183 µM) proved to be in the same range as the reported dissociation constants obtained with SPR (Table 1; Figure 2). Compound 24123034 demonstrated a similar AcrB binding affinity (601 µM), as determined via SPR. However, MST measurements revealed a four-fold stronger interaction of 36287038 with AcrB_His_ (402 µM) than previously reported. In addition, we assessed unspecific protein binding of the compounds by testing for interaction with His-tagged protease from tobacco etch virus (TEVp_His_). The protein was chosen due to its low sequence identity with AcrA_His_ (15.2%) and AcrB_His_ (21.4%). Notably, the binding affinity of 36287038 and 24123034 to TEVp was similar to the K_D_ values determined for AcrA_His_ and AcrB_His_. Compound 45, like 36287038, showed a stronger binding affinity to TEVp_His_ than to AcrA_His_.

To exclude the possibility that the compounds interact with the His-tag, the three compounds were tested for their ability to bind to a differently (GST) tagged protein (protease 3C). All three compounds demonstrated binding to _GST_3C. However, the dissociation constants were 1.5- to 3.5-fold higher compared to His-tagged AcrA and AcrB (Table 2). Compound 24123034 showed a higher affinity to _GST_3C than to AcrB_His_. This led to the interpretation that protein binding could be partially based on the His-tag, although binding to common structural features present in all tested proteins cannot be excluded.

### 3.4. Investigation of Colloidal Aggregate Formation

The binding of compounds 24123034, 36287038, and 45 to several proteins and little to no interaction with the tag prompted us to investigate whether this might be based on colloidal aggregation.

A web tool (https://admet.scbdd.com/ChemAGG/calc_cf_single_mol/; accessed on 10 January 2023) [26] to identify common structural elements of colloidal aggregators only identified compound 45 as a colloidal aggregator with a probability of 52%. In contrast, neither 24123034 (18%) nor 36287038 (23%) was predicted as a colloidal aggregator.

**Figure 3:**
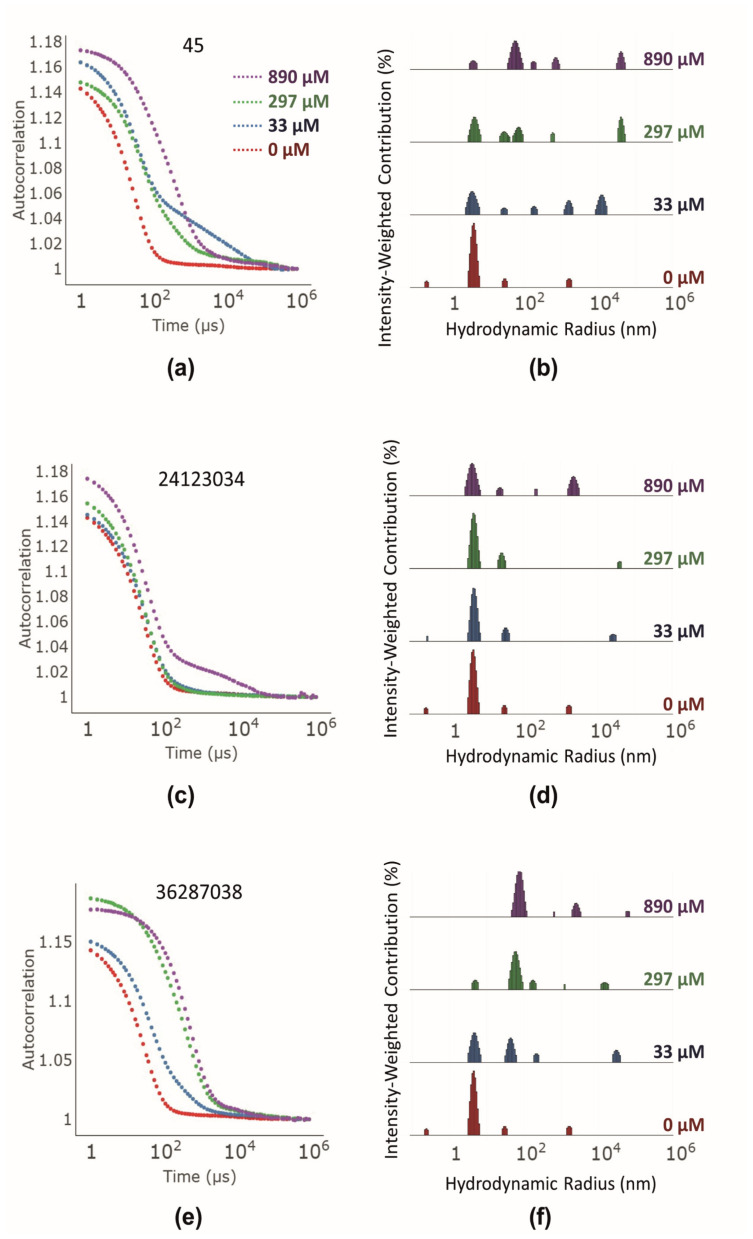
(**a**,**c**,**e**) Mean autocorrelation curves (*n* = 3) of compounds 45 (**a**), 24123034 (**c**), and 36287038 (**e**) at 0 µM (red), 33 µM (blue), 297 µM (green), and 890 µM (violet) measured using DLS (**b**,**d**,**f**). Derived histograms of compounds 45 (**b**), 24123034 (**d**), and 36287038 (**f**) using the online tool Raynals (https://spc.embl-hamburg.de/; accessed on 28 October 2022) [24].

DLS was used to determine whether the compounds formed aggregates [27]. Substances that form aggregates with hydrodynamic radii of 50 to 1000 nm are known to behave as colloidal aggregators [28]. Autocorrelation curves in the presence of AcrA indicated that compounds 45 (Figure 3a) and 36287038 (Figure 3e) formed 100 nm sized aggregates, while compound 24123034 (Figure 3c) formed bigger aggregates of approximately 1000 nm at the highest concentration (890 µM). The peaks for the relative number of formed aggregates measuring 100 nm and 1000 nm correlated with the tested compound concentration. Interestingly, the lowest aggregate-forming compound concentrations correspond to the measured K_D_ values. For example, compound 45 did not form 100 nm aggregates at a concentration below the K_D_ with AcrA_His_ (here 33 µM; Figure 3a,b). In contrast, compound 36287038 formed 100 nm aggregates at 33 µM (Figure 3e,f) and demonstrated a higher affinity to AcrA_His_ than compound 45 according to MST measurements. Compound 24123034 formed a significant number of larger aggregates at 890 µM (Figure 3c,d). In addition, we detected a small number of 100 nm sized aggregates at 33 µM and 297 µM. The binding affinity of 24123034 to AcrA_His_ did not correspond to the aggregate-forming concentrations. While a significant number of aggregates were formed at higher concentrations, the affinity to AcrA_His_ was modest (164 µM).

**Figure 4:**
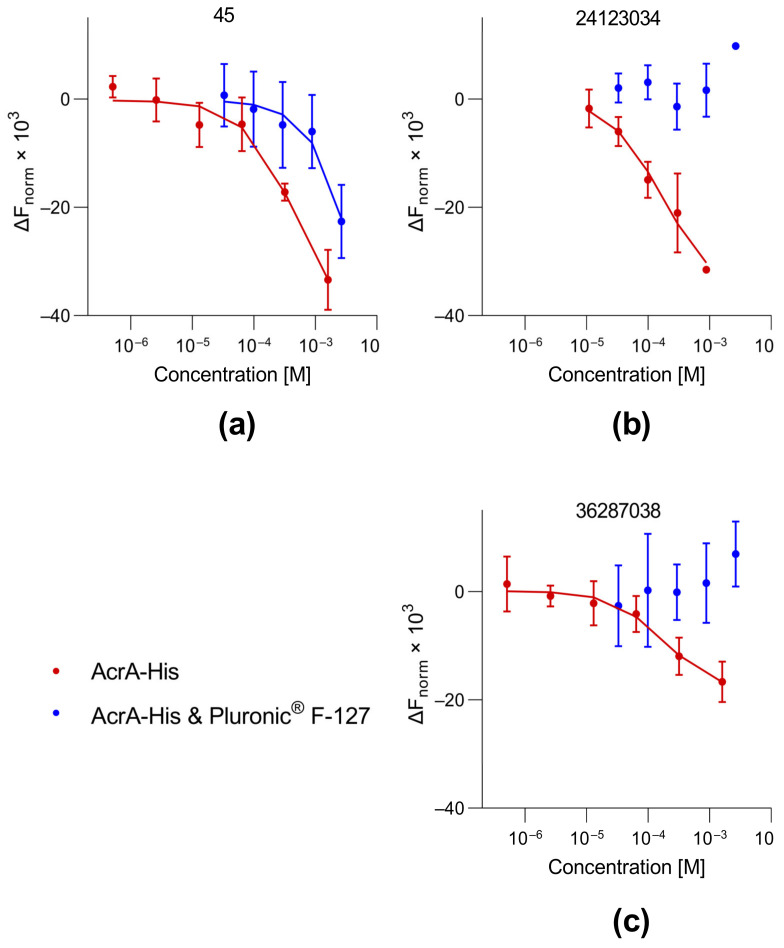
Dose–response curves obtained through MST using His-tagged AcrA without (red) and with addition of 0.08% Pluronic^®^ F-127 (blue) in the presence of compounds 45 (**a**), 24123034 (**b**), or 36287038 (**c**). To facilitate comparison of the binding data, the baseline was subtracted from the curve and multiplied by 1000. Each data point is presented as mean ± SD (*n* = 3). Data points were derived from Figures S17–S19.

Another commonly used method to identify colloidal aggregators is the addition of nonionic detergents to the assays. Although 0.05% of the nonionic detergent Tween^®^ 20 was already used to prevent interactions of proteins with the MST capillaries, binding to the aggregate-forming compounds still occurred. Previously, it has been observed in some cases that Tween^®^ 20 (0.005%) does not prevent binding of proteins to colloidal aggregators, as measured using SPR [29]. Thus, we added Pluronic^®^ F-127, a nonionic triblock copolymer. Those copolymers interact with solvent-exposed hydrophobic parts of unfolded proteins while not interacting with native proteins [30]. Colloidal aggregators interact with proteins by binding partially unfolded parts of a protein [31]. Pluronic^®^ F-127 should, in theory, compete with the compound aggregates by binding to partially unfolded proteins. Specific interactions between compounds and proteins should not be affected since those interactions occur with native proteins that are not bound by nonionic triblock copolymers. The decrease in affinity as well as the complete loss of binding/activity upon the addition of Pluronic^®^ F-127 is an indicator of nonspecific binding [27]. For compound 45, the addition of Pluronic^®^ F-127 (0.08%) led to a shift in binding to AcrA_His_ (Figure 4a). For the reference compounds 24123034 and 36287038, no binding to AcrA_His_ could be determined after the addition of Pluronic^®^ F-127 (Figure 4b,c). These results indicate that the binding to AcrA_His_ is nonspecific. In conjunction with DLS results, this proved that all synergistically acting compounds possessing a 4(3-aminocyclobutyl)pyrimidin-2-amine scaffold are colloidal aggregators. Therefore, all measured K_D_s should be considered apparent K_D_s.

As already mentioned before, aggregate binding to proteins stabilizes partial unfolding and thereby may result in a lower melting temperature (T_M_). Therefore, the T_M_ of AcrA_His_ was determined by means of nDSF in the presence and absence of compounds 45, 24123034, and 36287038. The T_M_ of AcrA_His_ in the absence of any compound was 50.4 °C. The addition of any of the three compounds at a concentration of 2 mM led to a negative T_M_ shift of AcrA_His_ (Table 3, Figures S21–S23).

To investigate whether the synergistic activity of the tested 4(3-aminocyclobutyl)pyrimidin-2-amines with erythromycin in the checkerboard assay was connected with the formation of aggregates, three compounds (2, 25, and 26) possessing structural attributes that were identified as inactive were tested using DLS (see Section 3.1 and Figure S3). The autocorrelation curves showed that compounds 2 and 26 also formed aggregates at 890 µM (Figure S20). The aggregates revealed hydrodynamic radii of roughly 1000 to 10,000 nm, and were thus bigger than the ones formed by the active compounds (Figure 3). Interestingly, compound 25, possessing a seven-membered diazacycloheptane instead of a piperidine, did not form any aggregates and had an autocorrelation curve similar to that of AcrA_His_ alone (Figure S20). In addition, the aforementioned nDSF measurements were repeated with the inactive compounds (Table 4). As expected, none of the compounds decreased the T_M_, thus indicating a lack of binding to partially unfolded proteins.

## 4. Discussion

AcrA represents an attractive target for efflux pump inhibitors as it is an essential part of the AcrAB-TolC complex. Compounds interfering with AcrA may act in multiple ways, for example, by interrupting protein-protein interactions with either AcrB or TolC, or preventing the assembly of the AcrA hexamer [32].

In order to expand the number of AcrA-targeting compounds, we searched a public database to identify other 4(3-aminocyclobutyl)pyrimidin-2-amines. This resulted in the identification of several commercially available compounds that improved the synergistic activity of erythromycin. In order to assess the binding affinity of these compounds to AcrA, MST was used. So far, investigations of small molecules binding to efflux pump proteins have been mainly restricted to SPR [33] or bio-layer interferometry (BLI) [34]. MST measurements correctly reproduced the K_D_ values for two of the previously reported 4(3-aminocyclobutyl)pyrimidin-2-amines, and thus represent an alternative approach for quantifying small-molecule binding to AcrA_His_. The binding studies revealed weak binding of the most potent compound, 45, to AcrA_His_ and AcrB_His_, similar to the previously reported molecules 24123034 and 36287038. In addition, all three substances also demonstrated binding to other proteins, such as TEVp and 3C.

In order to analyze the origin of this promiscuous interaction, we first investigated whether these compounds interfered with the His-tag. The previously reported binding affinities of 24123034 and 36287038 to AcrA, as determined using SPR, were obtained using His-tagged protein immobilized on a CM5 chip that interacts with proteins via -NH_2_, -SH, -CHO, -OH, or -COOH groups and not via a His-Tag. Thus, the His-tag was accessible in the reported SPR measurements, similar to our MST studies. However, the K_D_s obtained for GST-tagged 3C did not significantly differ from the His-tagged proteins, and therefore any compound interaction with the His-tag could be excluded.

The fact that the 4(3-aminocyclobutyl)pyrimidin-2-amines bound to four sequentially non-related proteins with similar binding constants (Table 1 and Table 2) raised the question of whether the binding could be of a nature not specific for AcrA or AcrB. Therefore, we investigated a common interference mechanism in high-throughput screening: colloidal aggregation. In this case, the compounds form aggregates 100–1000 nm in size in a dose-dependent manner and bind to partially unfolded proteins. These interactions are commonly misinterpreted as real binding events [35]. There are algorithms for assessing the likelihood a substance will aggregate; however, the one used in this study did not predict the 4(3-aminocyclobutyl)pyrimidin-2-amines as aggregators. It has been reported that the algorithms result in a significant rate of false positives and false negatives and that for the correct identification of colloidal aggregators, experimental investigation is crucial [36].

In our study, the test compounds showed aggregation in DLS measurements at concentrations correlating to the K_D_. Furthermore, the addition of a nonionic copolymer led to a K_D_ shift to millimolar concentrations or a complete loss of binding, which is typical for most colloidal aggregators. These control experiments were not conducted in previous binding studies [15]. Our findings are supported by nDSF measurements, a method that has not been used for the identification of colloidal aggregators so far. In the presence of the test compounds, the melting temperature of AcrA_His_ was lowered, indicating partial protein unfolding. This strongly supports the hypothesis that partially unfolded proteins are stabilized when bound to a colloidal aggregator. As a consequence, it can be concluded that 4(3-aminocyclobutyl)pyrimidin-2-amines do not bind to any of the four postulated ligand-binding sites on AcrA [15]. As a control, we measured three compounds without a synergistic effect in the checkerboard assay. These did not negatively shift the T_M_ of AcrA_His_. Thus, it can be hypothesized that nonspecific protein binding by aggregates was responsible for the synergistic activity in the checkerboard assay. Contrary to this hypothesis is that the synergistic compound concentrations were below the concentrations at which aggregates were formed in the DLS measurements. It has to be noted that compound aggregation was not tested using checkerboard assay conditions; therefore, it is not certain that aggregates were present at the tested concentrations. However, the checkerboard assay was conducted without detergent and with less organic solvent (DMSO). Since the formation of aggregates is dependent on the solubility of the compounds [27], this leads to the hypothesis that aggregates could have been formed at lower concentrations. Another aspect contrary to the hypothesis is that in previous studies, the compounds did not potentiate the activity of antibiotics in an efflux-deficient TolC mutant *E. coli* strain [15]. However, in our experiments the compounds did potentiate antibiotics in AcrAB-deficient *E. coli* and another TolC-deficient strain [37] (Table S7). These mutant strains are not commonly used in antimicrobial potentiation assays but did show efflux deficiency due to increased sensitivity to erythromycin under the same conditions as the wildtype strain (64-fold decrease in MIC). It is possible that the formerly reported TolC mutants were not sensitive to the effect of colloidal aggregators. A potential reason for this could be the compromised membrane integrity in TolC mutants [38], which could vary depending on the type of mutation. Compounds inactive in the checkerboard assay either did not form aggregates (compound 25) or had much larger hydrodynamic radii (approximately 10^4^ nm for compound 26). The most striking difference between the active and inactive compounds in the checkerboard assay was that the inactive compounds did not interact with AcrA_His_ in the nDSF measurements. This indicated that these compounds did not affect the protein fold, although aggregates might have been formed. Previous studies have shown that different aggregate-forming compounds exhibit different affinities for proteins [39]. Thus, it is possible that the aggregates of inactive compounds could also interact with the proteins, but not at the tested concentrations.

**Figure 5:**
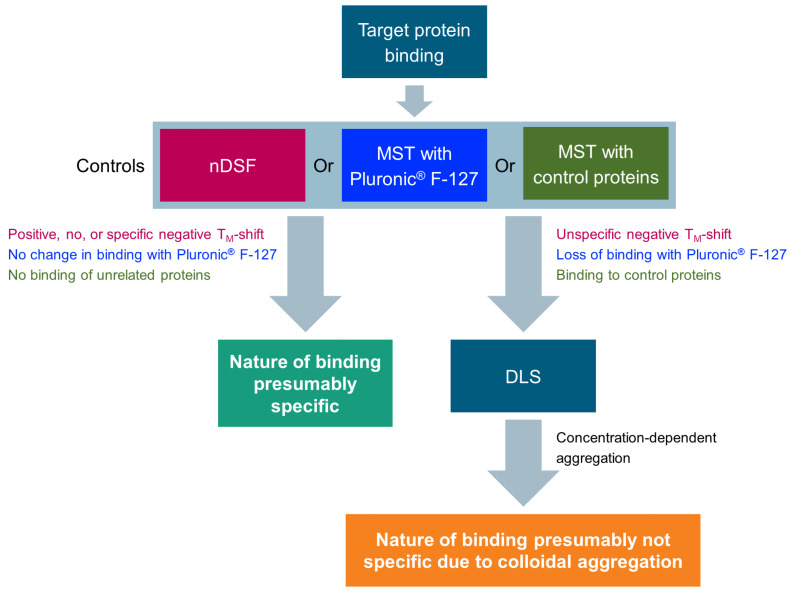
Suggested workflow for avoiding false positives due to colloidal aggregation when using MST measurements in drug discovery.

In drug discovery, screening methods are used to identify molecules with certain biological activity in a large library of chemically diverse compounds. Various options for counter screens exist, and strategies to avoid compound aggregation are frequently applied to eliminate false-positive hits [27,40]. It has been previously reported that colloidal aggregation can be identified using SPR, either if the curves look unusual or if any binding is lost upon addition of a nonionic detergent [29]. However, not all colloidal aggregators can be ruled out with this method. Our study shows that MST measurements also detect binding to colloidal aggregates. In our case, 0.05% Tween^®^ 20 was not sufficient to prevent the binding to proteins, but the addition of 0.08% Pluronic^®^ F-127 was. Our study shows the importance of implementing corresponding strategies for reproducing published binding data obtained by means of biophysical methods. We used a combination of assays to rule out nonspecific binding (Figure 5). Amongst the already-reported detergent sensitivity and formation of aggregates [27], negative T_M_ shifts in nDSF measurements can be a hint for interactions with colloidal aggregators. nDSF measurements are quick and often carried out in parallel to MST measurements as an orthogonal binding assay. If a compound shows binding to several proteins in biophysical binding assays and a small negative T_M_ shift in nDSF measurements, DLS measurements should be conducted to rule out binding that is mediated by colloidal aggregates. Alternatively, counter screens with Pluronic^®^ F-127 can be carried out.

## 5. Conclusions

A comprehensive investigation of 4(3-aminocyclobutyl)pyrimidin-2-amines revealed the compounds to have synergistic effects with erythromycin against *E. coli*. However, the mechanism of action of these substances is unclear as these were identified as colloidal aggregators that interact with proteins in a nonspecific manner. Our study highlights the importance of incorporating controls for biophysical methods, in particular for substances with binding affinities in the mid-micromolar to low-millimolar range.

## Figures and Tables

**Table 1:** Binding affinities (K_D_s and 95% confidence interval) of test compounds for His-tagged proteins AcrA and AcrB as determined through MST and SPR [15], as well as for the control protein TEVp_His_.

Cpd.	AcrA_His_ (MST) (µM) ^a^	AcrB_His_ (IF) (µM) ^b^	AcrA_His_ (SPR) (µM)	AcrB_S1043_ (SPR) (µM)	TEVp_His_ (MST) (µM) ^c^
45	485 (204; 1440)	301 (44.6; 1270)	N/A	N/A	174 (71.8; 422)
24123034	164 (42.4; 761)	601 (475; 718)	420	440	623 (23.9; 4550)
36287038	183 (46.3; 762)	402 (322; 493)	230	1610	88.6 (11; 603)

K_D_: dissociation constant, CI: confidence interval. ^a^ MST of 5 µM AcrA_His_ in the presence of test compounds at 14 to 15 s laser on-time for 36287038, 19 to 20 s for 24123034, and 9 to 10 s for 45. ^b^ Initial fluorescence (IF) quenching of 1.25 µM AcrB_His_ in the presence of test compounds before heating. Fitted curves are provided in Figure 2d. ^c^ MST of 5 µM TEVp_His_ in the presence of test compounds at 14 to 15 s laser on-time for 36287038, 19 to 20 s for 24123034, and 9 to 10 s for 45.

**Table 2:** Binding affinities for the GST-tagged protein 3C as determined through MST.

Compound	MST (_GST_3C) ^a^	
	K_D_ (µM)	CI 95%
45	903	582; 1420
24123034	334	111; 1070
36287038	619	231; 1700

^a^ MST using 5 µM _GST_3C in the presence of test compounds at 9 to 10 s laser on-time for 36287038, 0 to 1 s for 24123034, and 19 to 20 s for cpd. 45.

**Table 3:** Melting temperature of AcrA_His_ upon addition of the compounds, as determined by means of nDSF (*n* = 3). Melting temperatures were derived from Figures S21–S23.

Compound	T_M_ ±SD [°C] ^a^	C_measured_ (µM) ^b^
No compound	50.4 ± 0.0	-
45	49.5 ± 0.1	2000
24123034	50.0 ± 0.2	2000
36287038	49.8 ± 0.2	2000

^a^ T_M_: melting temperature. ^b^ C_measured_: compound concentration added to 5 µM AcrA_His._

**Table 4:** Melting temperature of AcrA_His_ after addition of the inactive compounds determined using nDSF (*n* = 3). Melting temperatures were derived from Figures S24–S26.

Compound	T_M_ ± SD [°C] ^a^	C_measured_ (µM) ^b^
No compound	50.2 ± 0.1	-
2	50.1 ± 0.1	1000 ^c^
25	50.1 ± 0.0	2000
26	50.1 ± 0.0	2000

^a^ T_M_: melting temperature. ^b^ C_measured_: test compound concentration added to 5 µM AcrA_His_. ^c^ Highest possible concentration due to low solubility.

## Data Availability

The data presented in this study are available on request from the corresponding author.

## Supplementary Materials

The following supporting information can be downloaded at: https://www.mdpi.com/article/10.3390/biom13061000/s1. Figure S1: melting curves of AcrA_His_ in buffer with different DMSO concentrations; Figure S2: turbidity measurements of the nDSF samples. Table S1: structure, MPC_4_, and MIC of control compounds; Table S2: structure, MPC_4_, and MIC of compounds modified at the cyclobutane substituent; Table S3: structure, MPC_4_, and MIC of compounds modified at the pyrimidine substituent; Table S4: structure, MPC_4_, and MIC of compounds modified at the piperidine; Table S5: structure, MPC_4_, and MIC of compounds modified at the piperidine substituent; Table S6: structure, MPC_4_, and MIC of compounds modified at the linker between piperidine and substituent. Figure S3: overview of most potent compounds with an MPC_4_ ranging from 12.5 to 100 µM; Table S7: MPC_4_ and MIC of selected compounds in efflux-deficient strains; Table S8: cell viability tests of selected compounds using the CellTiterGlo^®^ assay; Figure S4: dose–response curves for compounds 24123034, 36287038, and 45 titrated against GST-tagged 3C protease by MST; Figure S5: MST traces of 5 µM AcrA_His_ in the presence of different concentrations of compound 45; Figure S6: MST traces of 5 µM TEV_His_ in the presence of different concentrations of compound 45; Figure S7: MST traces of 5 µM AcrA_His_ in the presence of different concentrations of compound 24123034; Figure S8,: MST traces of 5 µM TEV_His_ in the presence of different concentrations of compound 24123034; Figure S9: MST traces of 5 µM AcrA_His_ in the presence of different concentrations of compound 36287038; Figure S10: MST traces of 5 µM TEV_His_ in the presence of different concentrations of compound 36287038; Figure S11: non-normalized MST traces of 1.25 µM AcrB_His_ in the presence of different concentrations of compound 45; Figure S12: non-normalized MST traces of 1.25 µM AcrB_His_ in the presence of different concentrations of compound 24123034; Figure S13: non-normalized MST traces of 1.25 µM AcrB_His_ in the presence of different concentrations of compound 36287038; Figure S14: MST traces of 4 µM _GST_3c in the presence of different concentrations of compound 45; Figure S15: MST traces of 4 µM _GST_3c in the presence of different concentrations of compound 24123034; Figure S16: MST traces of 4 µM _GST_3c in the presence of different concentrations of compound 36287038; Figure S17: MST traces of 5 µM AcrA_His_ in the presence of 0.08% Pluronic^®^ F-127 and different concentrations of compound 45; Figure S18: MST traces of 5 µM AcrA_His_ in the presence of 0.08% Pluronic^®^ F-127 and different concentrations of compound 24123034; Figure S19: MST traces of 5 µM AcrA_His_ in the presence of 0.08% Pluronic^®^ F-127 and different concentrations of compound 36287038; Figure S20: DLS measurements of inactive compounds; Figure S21: first derivative of the melting curve (nDSF) of 5 µM AcrA_His_ in the presence of 2 mM compound 45; Figure S22: first derivative of the melting curve (nDSF) of 5 µM AcrA_His_ in the presence of 2 mM compound 24123034; Figure S23: first derivative of the melting curve (nDSF) of 5 µM AcrA_His_ in the presence of 2 mM compound 36287038; Figure S24: first derivative of the melting curve (nDSF) of 5 µM AcrA_His_ in the presence of 1 mM compound 2; Figure S25: first derivative of the melting curve (nDSF) of 5 µM AcrA_His_ in the presence of 2 mM compound 25; Figure S26: first derivative of the melting curve (nDSF) of 5 µM AcrA_His_ in the presence of 2 mM compound 26. Table S1. Structure, MPC_4_ and MIC of control compounds [14]. Table S2. Structure, MPC_4_ and MIC of compounds modified at the cyclobutane substituent (compared to 36287038). Table S3. Structure, MPC_4_ and MIC of compounds modified at the pyrimidine substituent (compared to 36287038). Table S4. Structure, MPC_4_ and MIC of compounds modified at the piperidine (compared to 36287038). Table S5. Structure, MPC_4_ and MIC of compounds modified at the piperidine substituent (compared to 36287038). Table S6. Structure, MPC_4_ and MIC of compounds modified at the linker between piperidine and substituent (compared to 36287038). Table S7. MPC_4_ and MIC of selected compounds in efflux deficient strains. Table S8. Cell viability tests of selected compounds using the CellTiterGlo^®^ assay (CC50 ±SD).
