# Supplementary material for: Efflux Pump-Binding 4(3-Aminocyclobutyl)Pyrimidin-2-Amines Are Colloidal Aggregators
Source: Biomolecules. 2023 Jun 16;13(6):1000. doi: 10.3390/biom13061000 (PMC10296211; doi:10.3390/biom13061000)

Supplementary Material

Efflux Pump-Binding 4(3-Aminocyclobutyl)Pyrimidin-2-Amines Are Colloidal Aggregators

Tania Szal [1,2], Shweta Singh Chauhan [3,4], Philipp Lewe [5], Fatima-Zahra Rachad [1], Marina Madre [6], Laura Paunina [6], Susanne Witt [5], Ramakrishnan Parthasarathi [3,4] and Björn Windshügel [1,2,*]

[1] Fraunhofer Institute for Translational Medicine and Pharmacology ITMP, Discovery Research ScreeningPort, 22525 Hamburg, Germany; tania.szal@itmp.fraunhofer.de (T.S.); fatima-zahra.rachad@itmp.fraunhofer.de (F.-Z.R.)
[2] School of Science, Constructor University, 28759 Bremen, Germany
[3] Computational Toxicology Facility, Toxicoinformatics & Industrial Research CSIR-Indian Institute of Toxicology Research, Vishvigyan Bhavan, 31, Mahatma Gandhi Marg, Lucknow 226001, Uttar Pradesh, India; shwetasingh1chauhan@gmail.com (S.S.C.); partha.ram@iitr.res.in (R.P.)
[4] Academy of Scientific and Innovative Research (AcSIR), Ghaziabad 201002, Uttar Pradesh, India
[5] Centre for Structural Systems Biology (CSSB), University Medical Center Hamburg-Eppendorf (UKE), 22607 Hamburg, Germany; philipp.lewe@cssb-hamburg.de (P.L.); susanne.witt@cssb-hamburg.de (S.W.)
[6] Latvian Institute of Organic Synthesis, LV-1006 Riga, Latvia; madre@osi.lv (M.M.); laurapaunina@gmail.com (L.P.)
* Correspondence: bjoern.windshuegel@itmp.fraunhofer.de; Tel.: +49-40-303764-286

Figure S1. Melting curves of $AcrA_{His}$ in buffer at different DMSO concentrations.

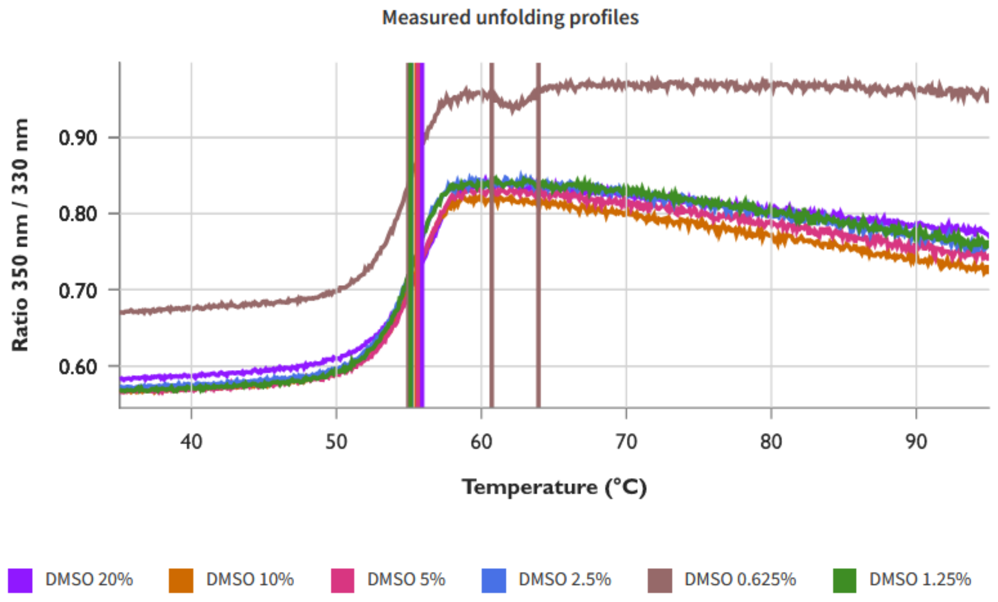

Figure S2. Scattering curves of $AcrA_{His}$ in presence of test compounds.

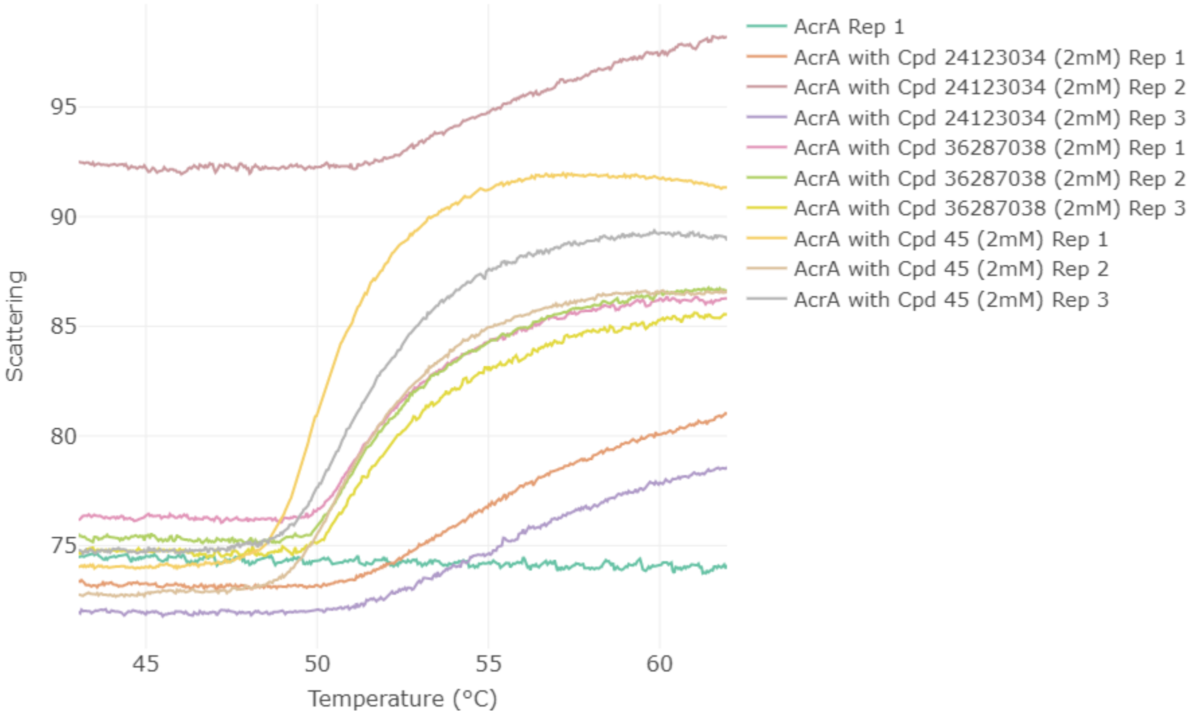

Table S1. Structure, MPC_4 and MIC of control compounds [14].

Compound	Structure	MPC_4 ±SD [µM]	MIC ±SD [µM]
**36287038**	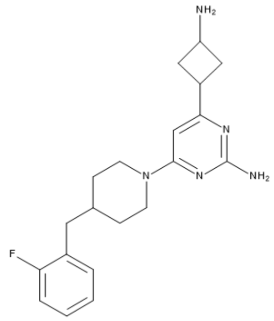	20.8 ±5.9	400 ±0.0
**24123034**	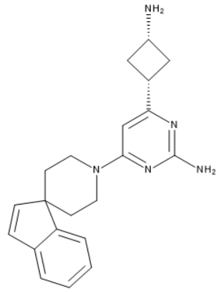	25 ±0.0	>50

Table S2. Structure, MPC_4 and MIC of compounds modified at the cyclobutane substituent (compared to 36287038).

Compound	Structure	MPC_4 [µM]	MIC [µM]
**1**	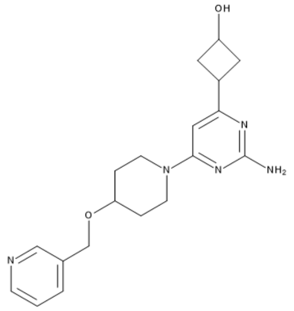	>400	>400
**2**	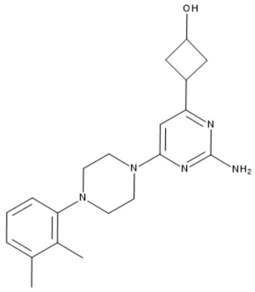	>400	>400
**3**	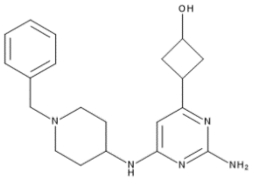	>100	>100
**4**	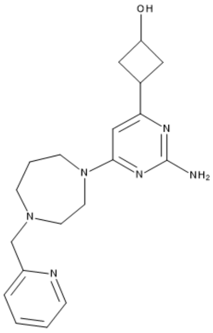	>100	>100
**5**	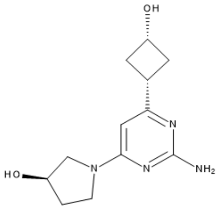	>400	>400
**6**	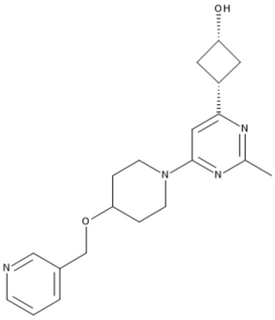	>400	>400

**7**	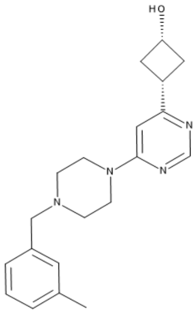	>400	>400
**8**	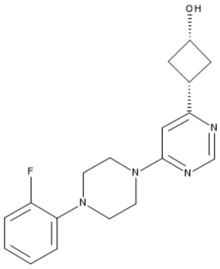	>400	>400
**9**	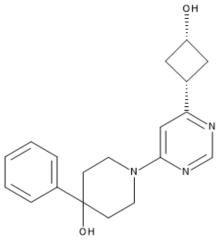	>400	>400
**10***	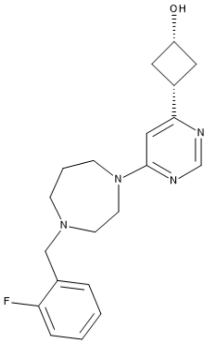	>400	>400

*Compound 10 was used as monohydrochloride salt.

Table S3. Structure, MPC_4 and MIC of compounds modified at the pyrimidine substituent (compared to 36287038)

Compound	Structure	MPC_4 [µM]	MIC [µM]
**11***	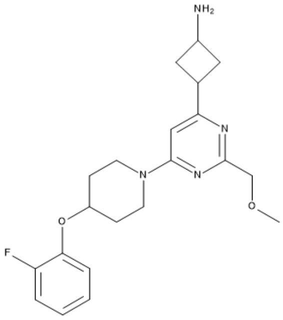	>400	>400
**12***	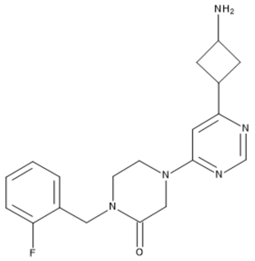	>400	>400

*Compound 11 was used as monohydrochloride salt and compound 12 as dihydrochloride salt.

Table S4. Structure, MPC_4 and MIC of compounds modified at the piperidine (compared to 36287038)

Compound	Structure	MPC_4 ± SD [µM]	MIC ± SD [µM]
**13**	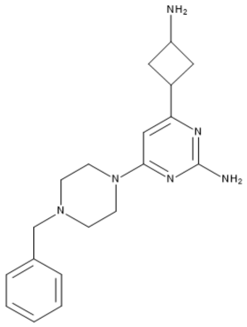	400 ±0.0	>400
**14**	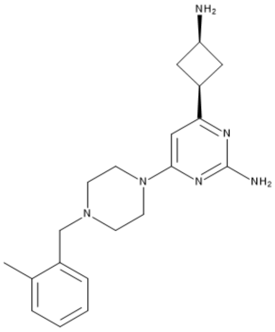	100 ±0.0	>400
**15**	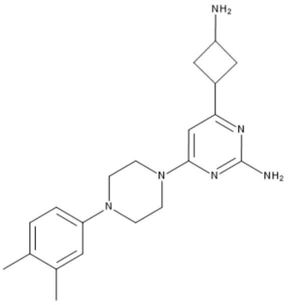	33.3 ±12	400 ±0.0
**16***	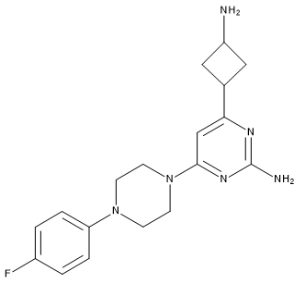	200 ±0.0	>400
**17**	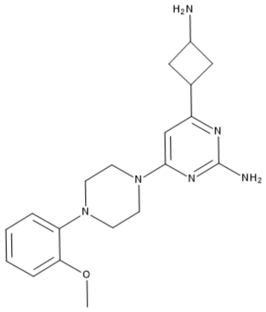	200 ±0.0	>400

**18***	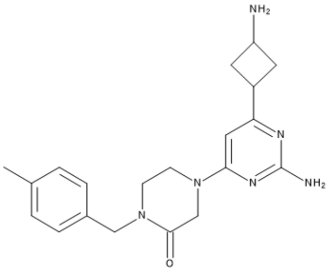	333 ±94	>400
**19***	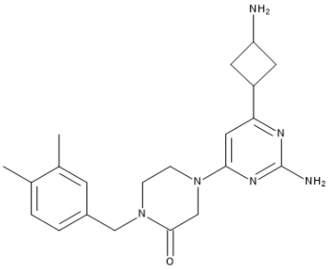	100 ±0.0	>400
**20**	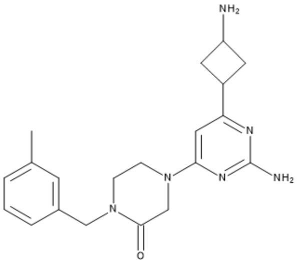	267 ±94	>400
**21**	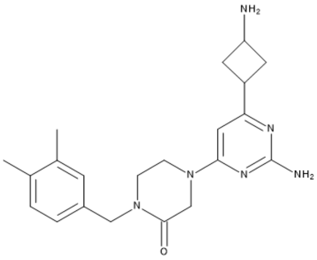	133 ±47	>400
**22**	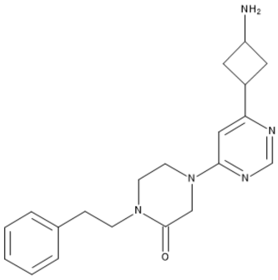	>400	>400
**23***	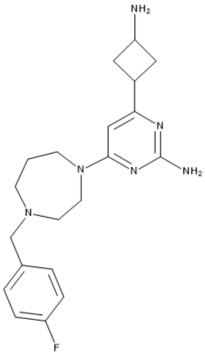	333 ±94	>400

**24**	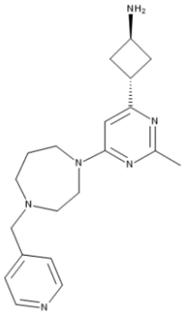	>400	>400
**25**	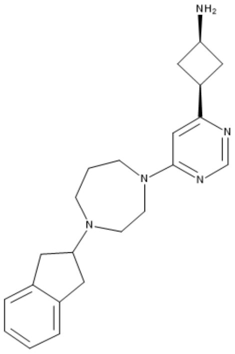	>400	>400
**26**	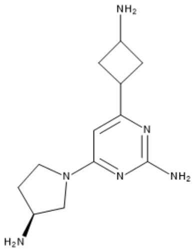	>400	>400
**27**	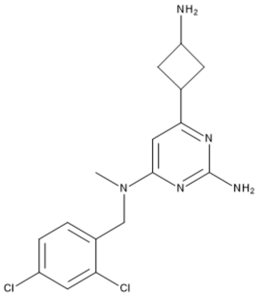	25.0 ±0.0	>100
**28**	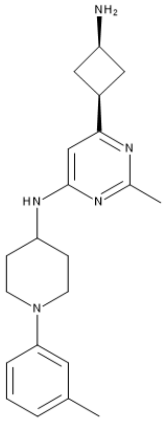	333 ±94	>400

*Compounds 16, 18, 19 and 23 were used as as dihydrochloride salts.

Table S5. Structure, MPC_4 and MIC of compounds modified at the piperidine substituent (compared to 36287038)

Compound-Nr.	Structure	MPC_4 ± SD [µM]	MIC ± SD [µM]
**29***	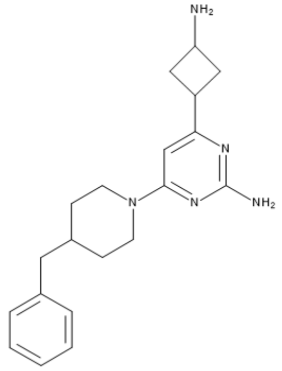	25.0 ±0.0	333 ±94
**30**	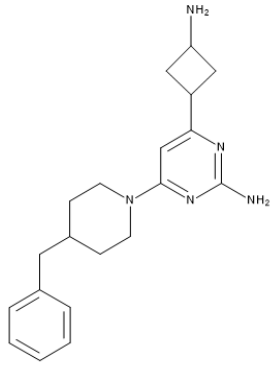	50.0 ±0.0	>400
**31**	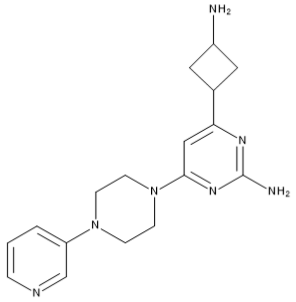	>400	>400
**32**	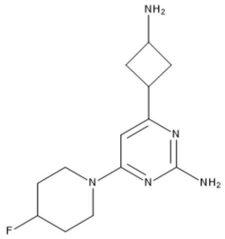	>400	>400
**33**	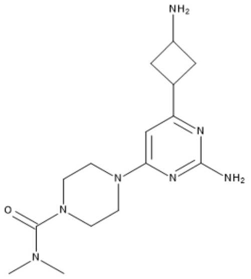	>400	>400

**34**	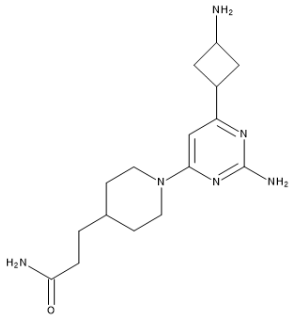	>400	>400

*Compound 29 was used as dihydrochloride salt.

Table S6. Structure, MPC_4 and MIC of compounds modified at the linker between piperidine and substituent (compared to 36287038)

Compound	Structure	MPC_4 ± SD [µM]	MIC ± SD [µM]
**35**	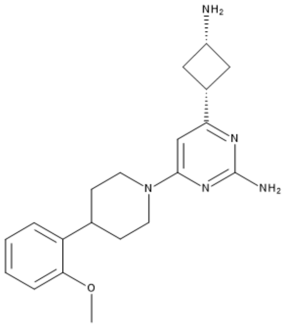	25.0 ±0.0	>400
**36**	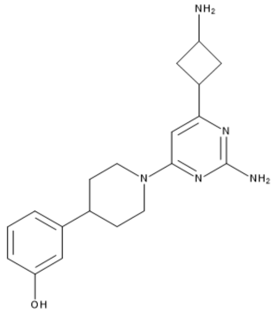	83.3 ±24	>400
**37**	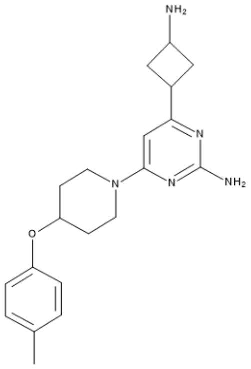	33.3 ±12	>400
**38***	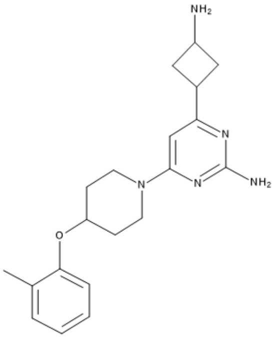	33.3 ±0.0	400 ±0.0
**39**	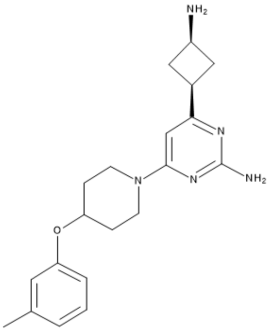	41.7 ±12	>100

**40***	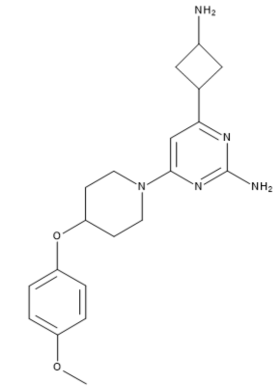	100 ±0.0	> 400
**41***	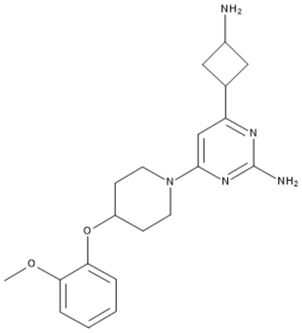	133 ±47	> 400
**42***	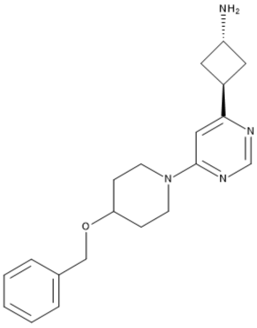	>400	> 400
**43**	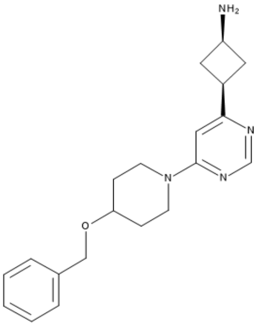	>400	> 400
**44**	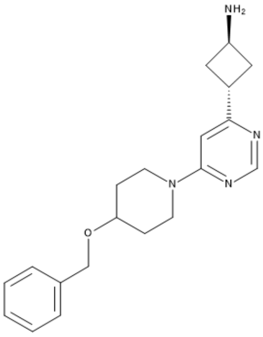	>100	>100

**45***	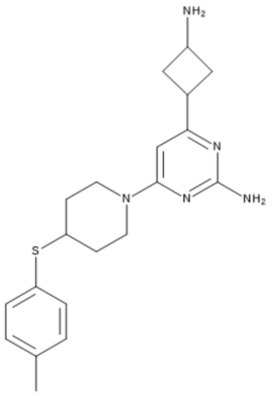	12.5 ±0.0	200 ±0.0
**46**	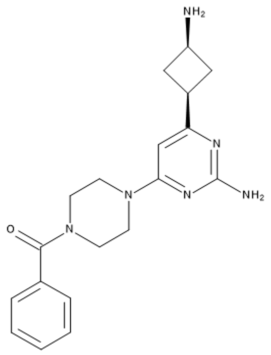	>400	> 400
**47**	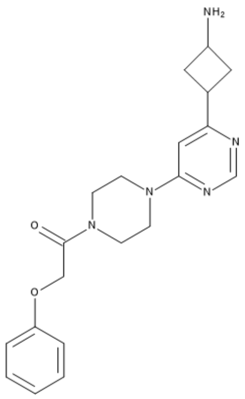	> 100	>100
**48**	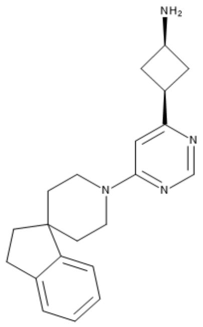	>100	>100

*Compounds 38, 40, 41, 42, and 45 were used as dihydrochloride salts.

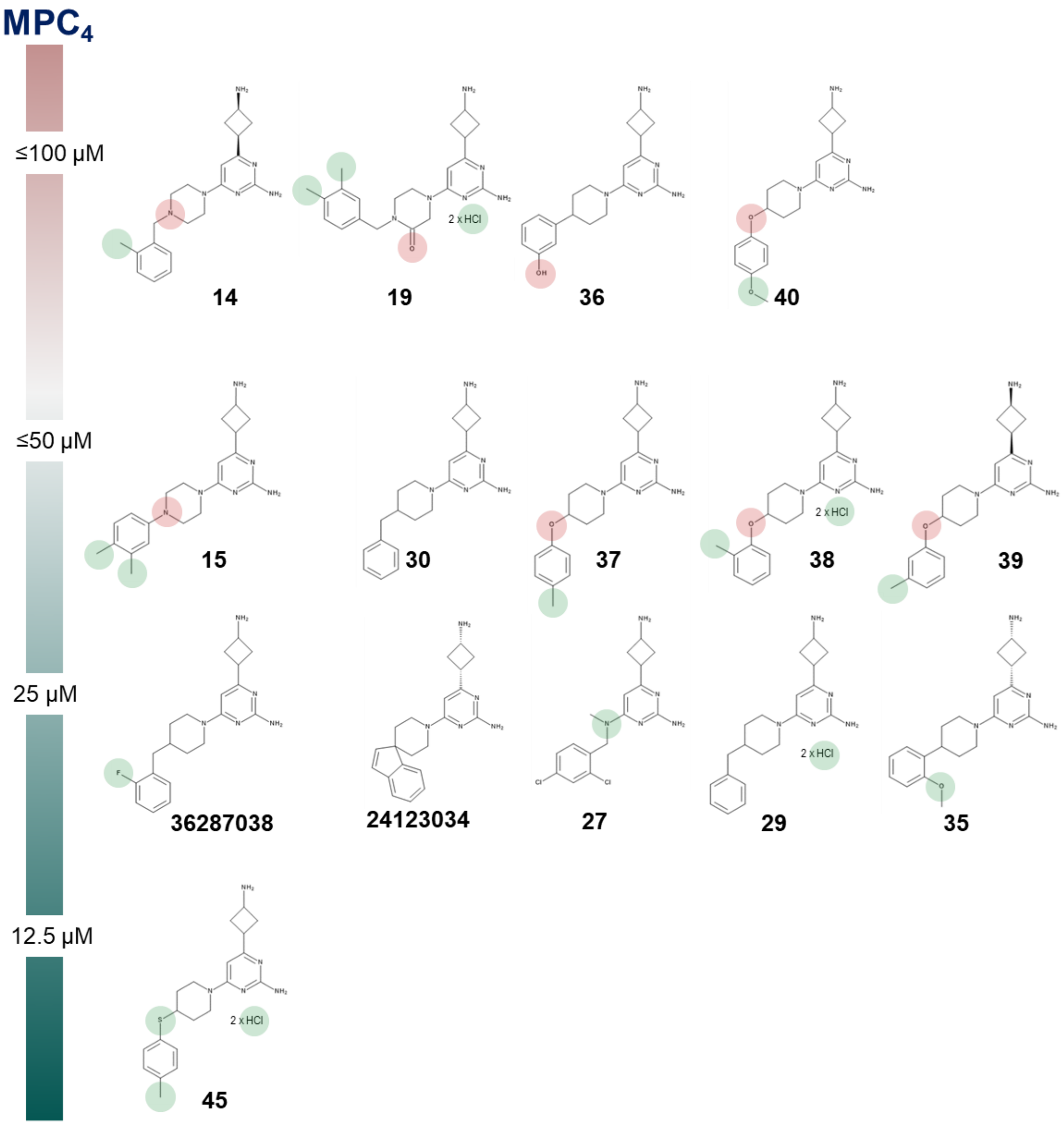

Figure S3. Overview of most potent compounds with a MPC$_4$ ranging from 12.5 to 100 µM. Marked in red are structural differences compared to the reference structure that indicate a decrease in synergistic activity, while the substructures highlighted in green resulted in an increase in synergistic activity as described in the text. Compounds 19, 29, 38, 40 and 45 were used as salts (mono- and dihydrochlorides).

Table S7. MPC_4 and MIC of selected compounds in efflux deficient strains

	ΔAcrAB		ΔTolC	
**Compound**	**MPC_4 [µM]**	**MIC [µM]**	**MPC_4 [µM]**	**MIC [µM]**
**45**	<25	>50	<25	>50
**36287038**	<25	>50	<25	>50
**24123034**	<25	>50	<25	>50

Table S8. Cell viability tests of selected compounds using the CellTiterGlo® assay (CC_{50} ±SD).

Compound	HepG2 [µM]	A549 [µM]	HEK-293 [µM]
**29**	29.2 ±4.2	26.7 ±3.2	17.2 ±2.2
**35**	27.0 ±0.8	27.4 ±0.7	19.7 ±2.8
**38**	11.0 ±2.8	12.5 ±1.8	7.3 ±0.3
**45**	9.4 ± 1.4	14.7 ± 2.2	12.7 ± 2.6

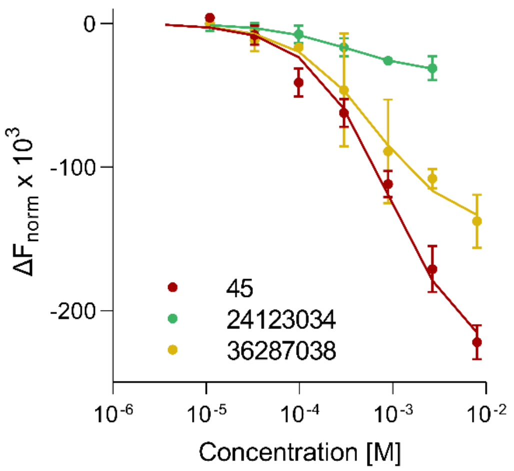

Figure S4. Dose-response curves for compounds 24123034 (green), 36287038 (yellow) and 45 (red) titrated against GST-tagged 3C protease by microscale thermophoresis. Curves are based on the normalized fluorescence (F_{Norm}) obtained at certain time points (Table 2). To facilitate comparison the difference of each concentration and the signal of protein without compound was plotted and multiplied by 1000. Each data point is presented as mean ± SD (n=3). The resulting dose-response curves were fitted to a one-site binding model by ThermoAffinity (https://spc.embl-hamburg.de/) to extract K_D values. Data points were derived from figures S13-S15.

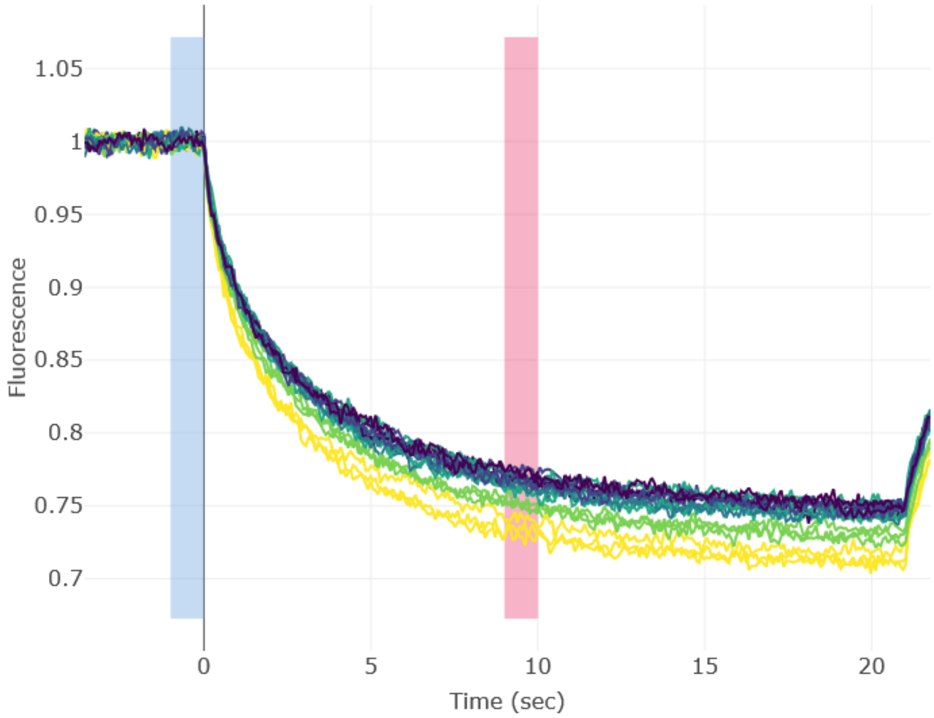

Figure S5. MST traces of 5 µM $AcrA_{His}$ in presence of different concentrations of compound 45 (n=3). The lighter colours (yellow and green) indicate higher concentrations (1600 µM to 64 µM compound concentration), while the darker colours (blue, violet and black) indicate lower concentrations (12.8 to 0.51 µM). The blue column marks the time range of the cold region and the red one marks the one selected for the hot region.

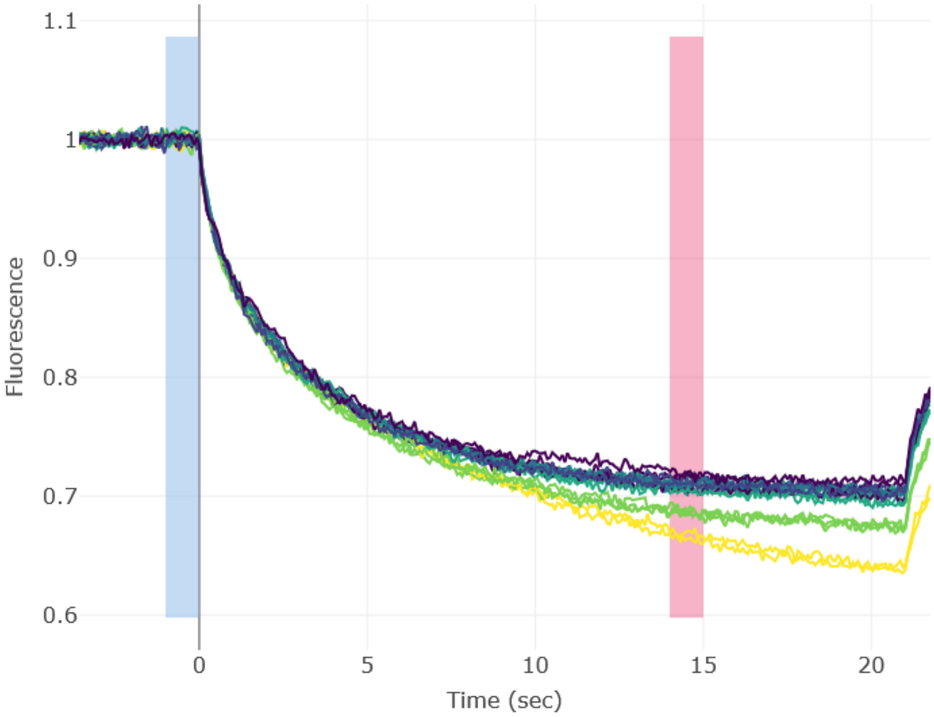

Figure S6. MST traces of 5 µM TEV_{His} in presence of different concentrations of compound 45 (n=3). The lighter colours (yellow and green) indicate higher concentrations (1600 µM to 64 µM compound concentration), while the darker colours (blue, violet and black) indicate lower concentrations (12.8 to 0.51 µM). The blue column marks the time range of the cold region and the red one marks the one selected for the hot region.

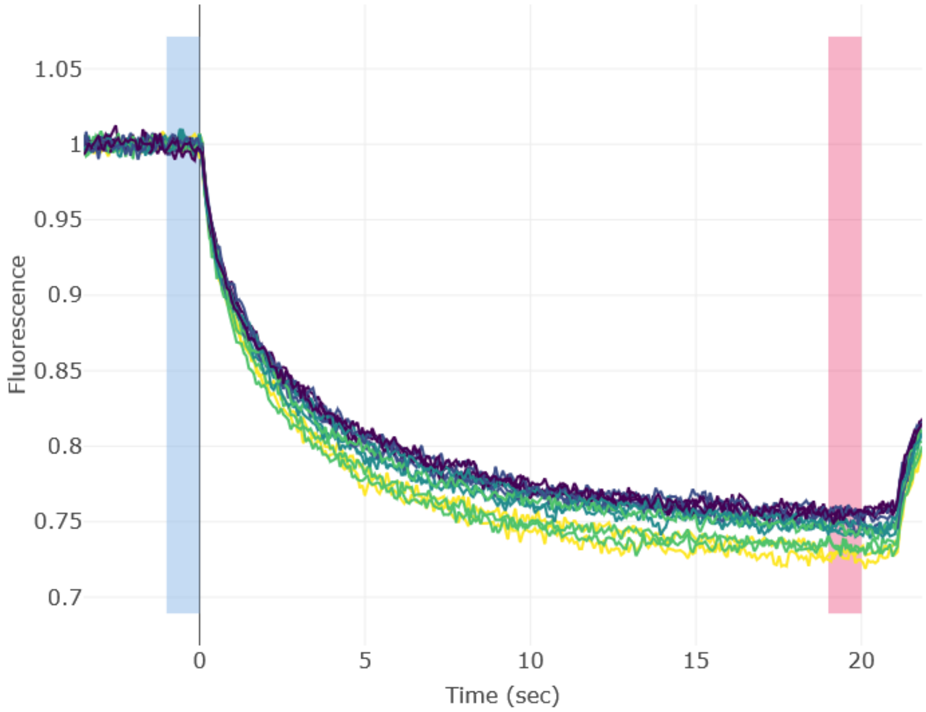

Figure S7. MST traces of 5 µM $AcrA_{His}$ in presence of different concentrations of compound 24123034 (n=3). The lighter colours (yellow and green) indicate higher concentrations (889 µM to 99 µM compound concentration), while the darker colours (blue, and violet) indicate lower concentrations (33 to 11 µM). The blue column marks the time range of the cold region and the red one marks the one selected for the hot region.

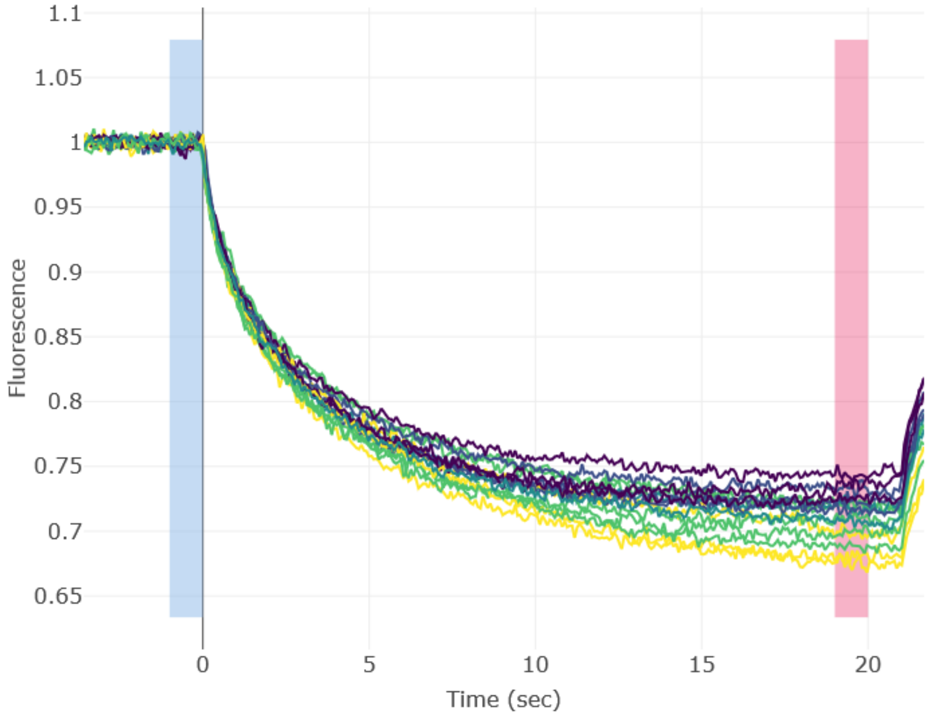

Figure S8. MST traces of 5 µM TEV_{His} in presence of different concentrations of compound 24123034 (n=3). The lighter colours (yellow and green) indicate higher concentrations (889 µM to 99 µM compound concentration), while the darker colours (blue, and violet) indicate lower concentrations (33 to 11 µM). The blue column marks the time range of the cold region and the red one marks the one selected for the hot region.

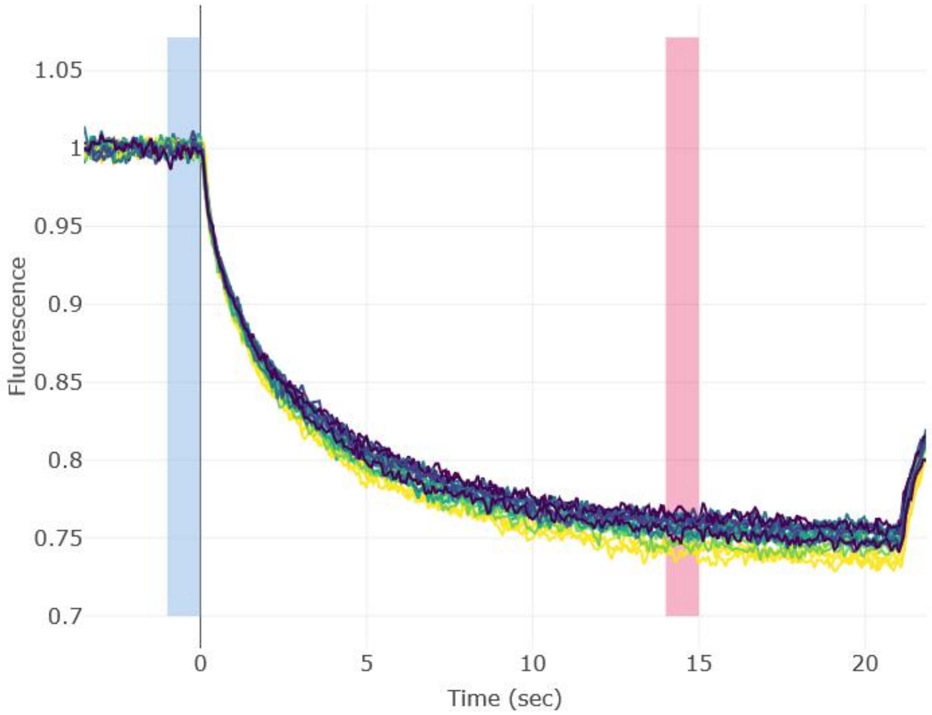

Figure S9. MST traces of 5 µM $AcrA_{His}$ in presence of different concentrations of compound 36287038 (n=3). The lighter colours (yellow and green) indicate higher concentrations (1600 µM to 64 µM compound concentration), while the darker colours (blue, violet and black) indicate lower concentrations (12.8 to 0.51 µM). The blue column marks the time range of the cold region and the red one marks the one selected for the hot region.

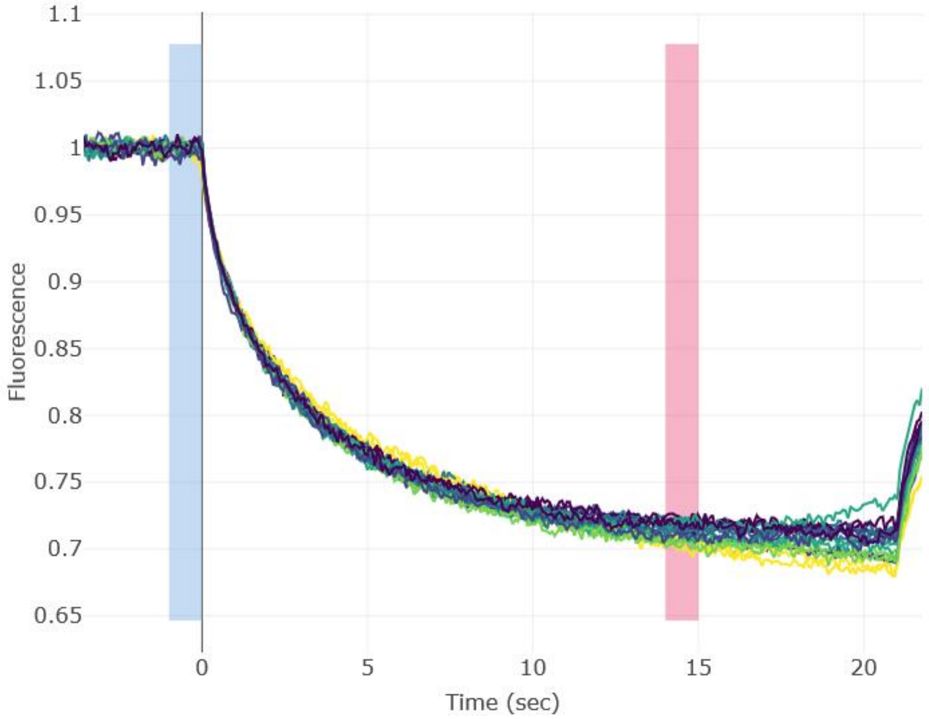

Figure S10. MST traces of 5 µM TEV_{His} in presence of different concentrations of compound 36287038 (n=3). The lighter colours (yellow and green) indicate higher concentrations (1600 µM to 64 µM compound concentration), while the darker colours (blue, violet and black) indicate lower concentrations (12.8 to 0.51 µM). The blue column marks the time range of the cold region and the red one marks the one selected for the hot region.

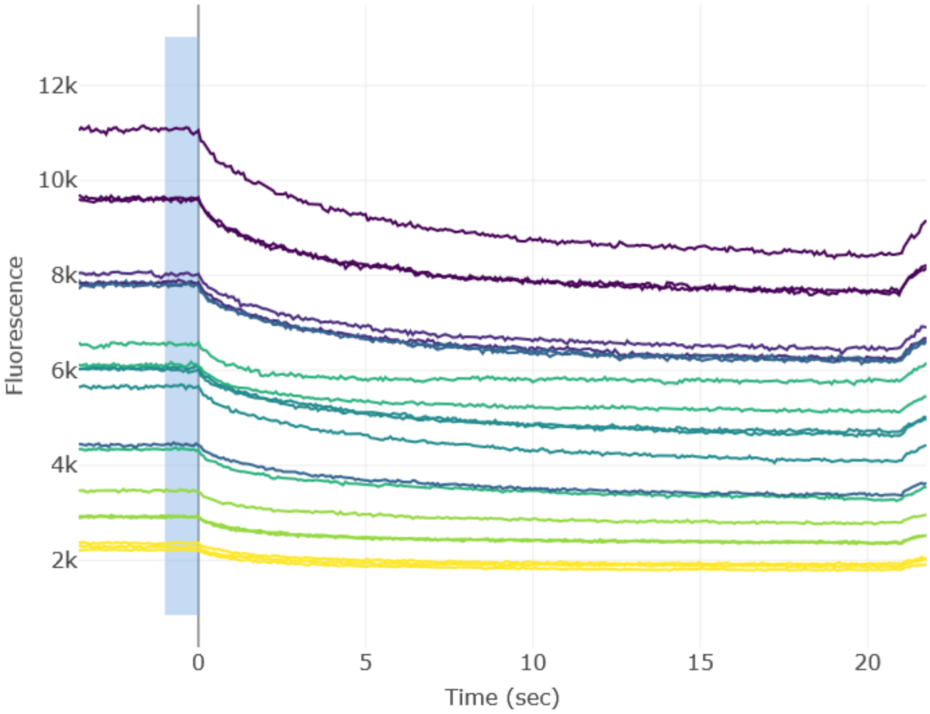

Figure S11. Not normalized MST traces of 1.25 µM $AcrB_{His}$ in presence of different concentrations of compound 45 (n=3). The lighter colours (yellow and green) indicate higher concentrations (8000 µM to 297 µM compound concentration), while the darker colours (blue, violet and black) indicate lower concentrations (99 to 11 µM).

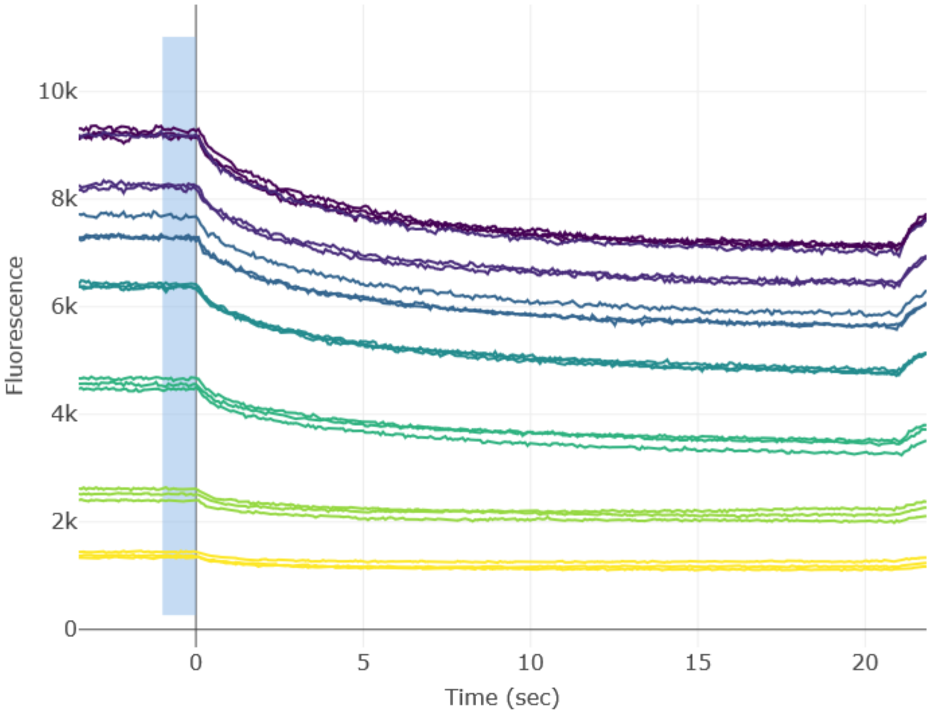

Figure S12. Not normalized MST traces of 1.25 µM $AcrB_{His}$ in presence of different concentrations of compound 24123034 (n=3). The lighter colours (yellow and green) indicate higher concentrations (8000 µM to 297 µM compound concentration), while the darker colours (blue, violet and black) indicate lower concentrations (99 to 11 µM).

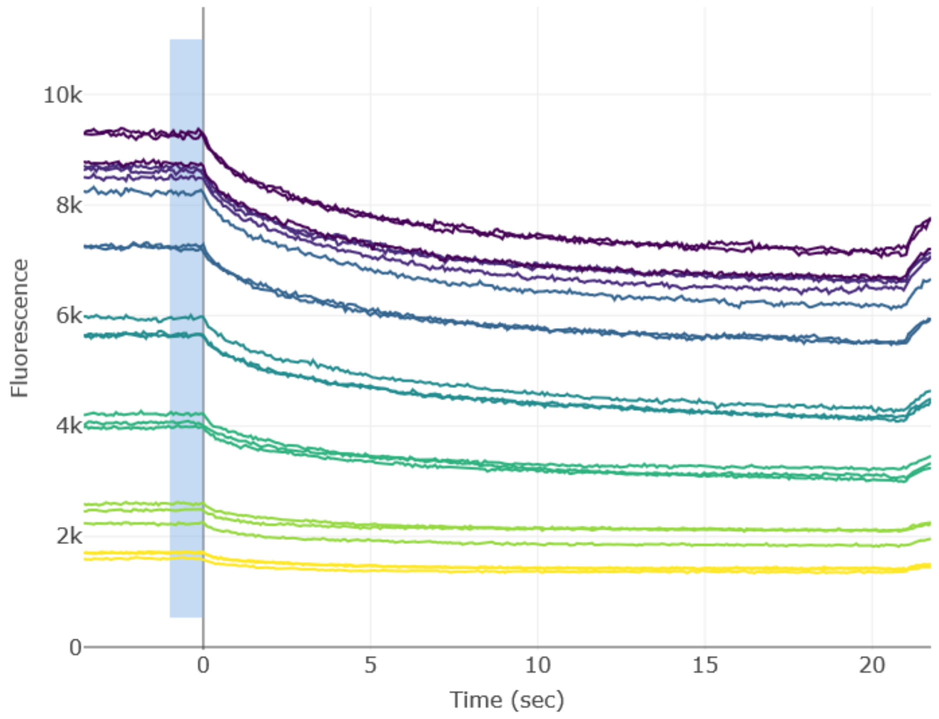

Figure S13. Not normalized MST traces of 1.25 µM AcrB$_{His}$ in presence of different concentrations of compound 36287038 (n=3). The lighter colours (yellow and green) indicate higher concentrations (8000 µM to 297 µM compound concentration), while the darker colours (blue, violet and black) indicate lower concentrations (99 to 11 µM).

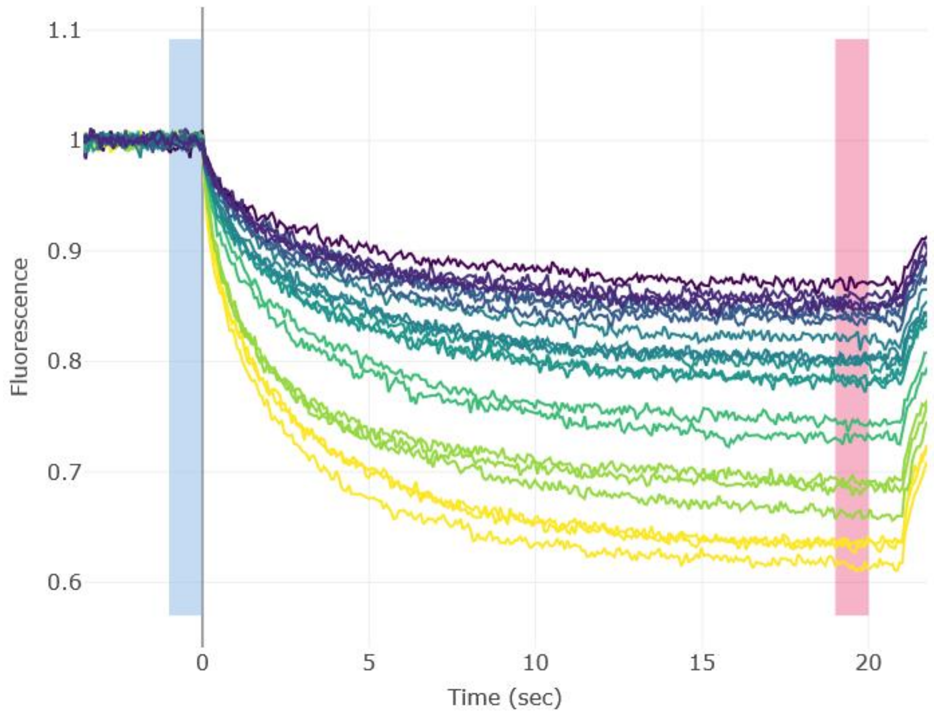

Figure S14. MST traces of 4 µM $_{GST}$3c in presence of different concentrations of compound 45 (n=3). The lighter colours (yellow and green) indicate higher concentrations (8000 µM to 297 µM compound concentration), while the darker colours (blue, violet and black) indicate lower concentrations (99 to 3.7 µM). The blue column marks the time range of the cold region and the red one marks the one selected for the hot region.

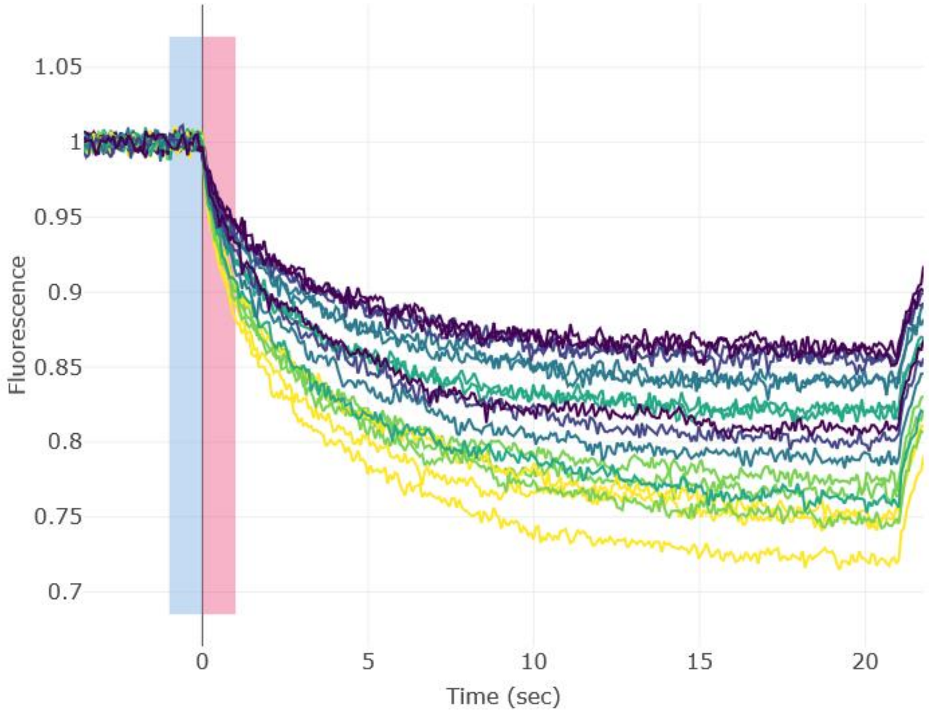

Figure S15. MST traces of 4 µM $_{GST}$3c in presence of different concentrations of compound 24123034 (n=3). The lighter colours (yellow and green) indicate higher concentrations (8000 µM to 297 µM compound concentration), while the darker colours (blue, violet and black) indicate lower concentrations (99 to 11 µM). The blue column marks the time range of the cold region and the red one marks the one selected for the hot region.

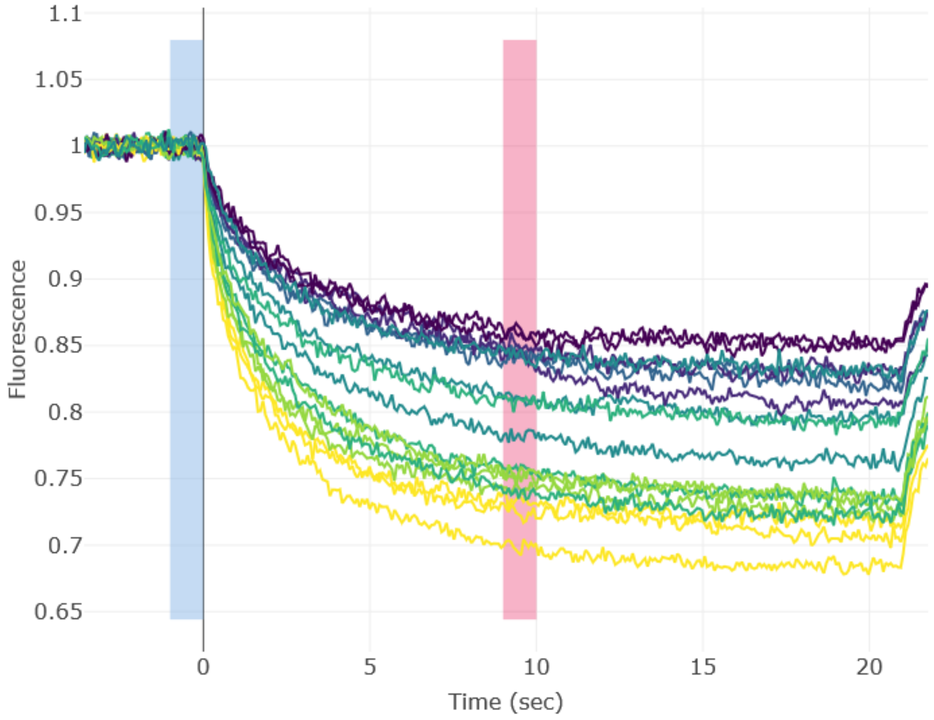

Figure S16. MST traces of 4 µM $_{GST}$3c in presence of different concentrations of compound 36287038 (n=3). The lighter colours (yellow and green) indicate higher concentrations (8000 µM to 297 µM compound concentration), while the darker colours (blue, violet and black) indicate lower concentrations (99 to 11 µM). The blue column marks the time range of the cold region and the red one marks the one selected for the hot region.

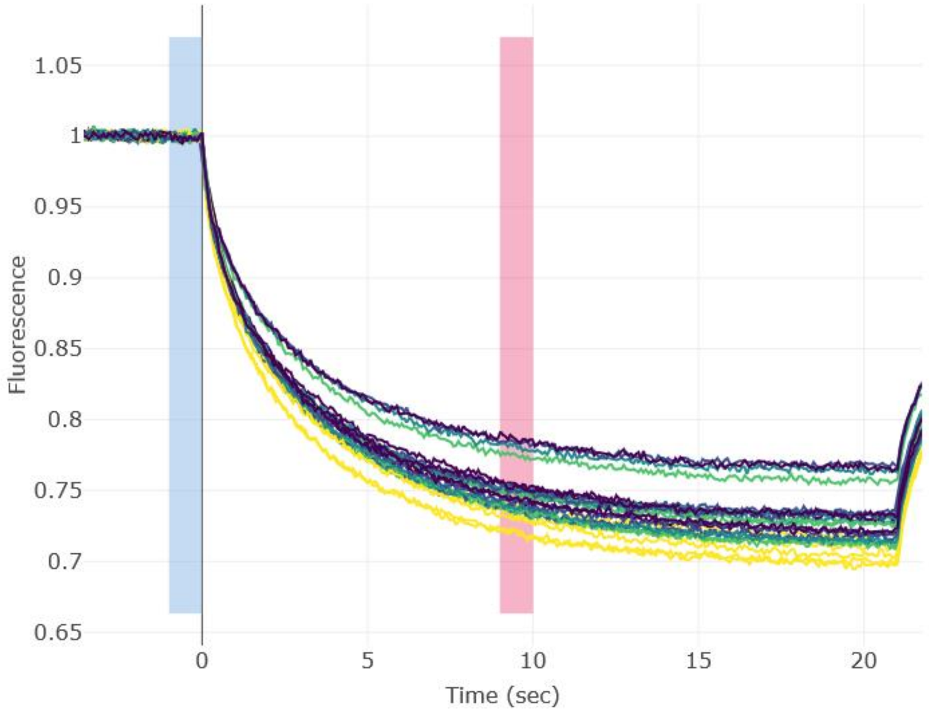

Figure S17. MST traces of 5 µM $AcrA_{His}$ in presence of 0.08% Pluronic F-127 and different concentrations of compound 45 (n=3). The lighter colours (yellow and green) indicate higher concentrations (2667 µM to 297 µM compound concentration), while the darker colours (blue, and violet) indicate lower concentrations (99 to 33 µM). The blue column marks the time range of the cold region and the red one marks the one selected for the hot region.

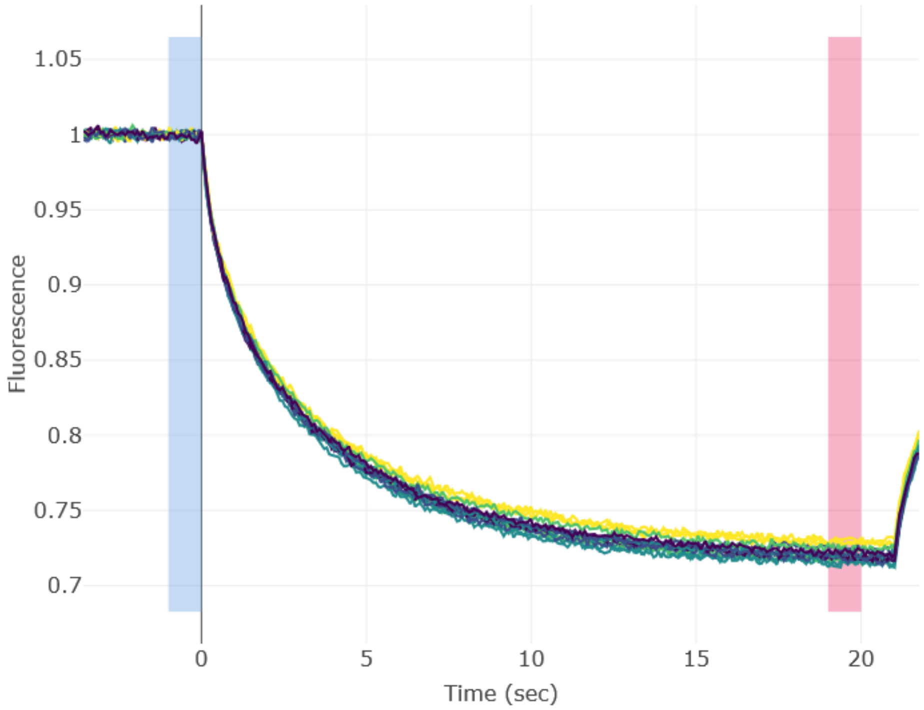

Figure S18. MST traces of 5 µM AcrA$_{His}$ in presence of 0.08 % Pluronic F-127 and different concentrations of compound 24123034 (n=3). The lighter colours (yellow and green) indicate higher concentrations (2667 µM to 297 µM compound concentration), while the darker colours (blue, and violet) indicate lower concentrations (99 to 33 µM). The blue column marks the time range of the cold region and the red one marks the one selected for the hot region.

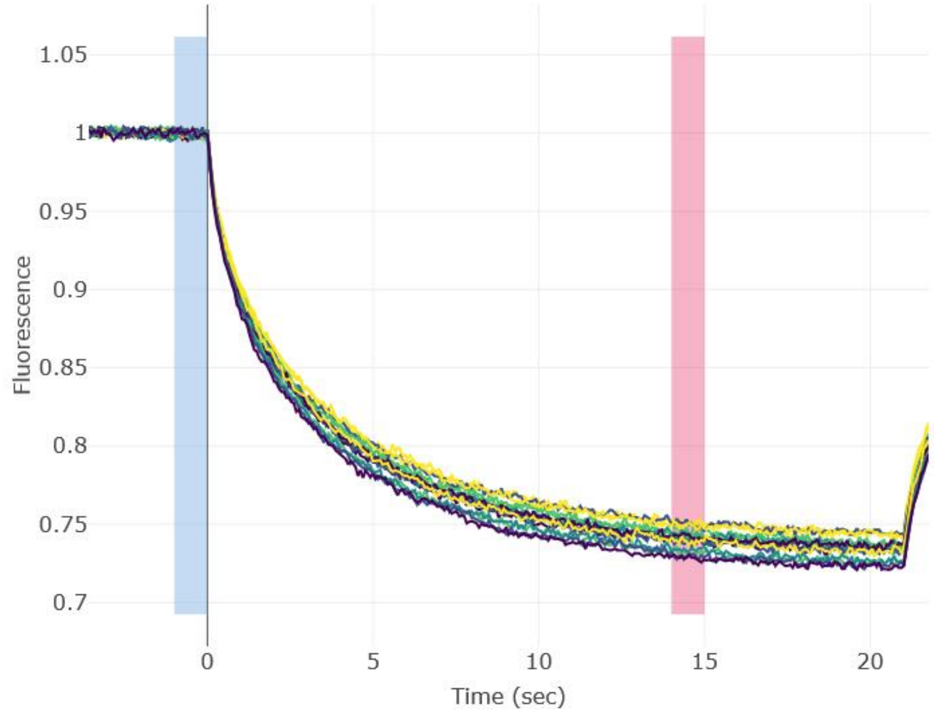

Figure S19. MST traces of 5 µM $AcrA_{His}$ in presence of 0.08 % Pluronic F-127 and different concentrations of compound 36287038 (n=3). The lighter colours (yellow and green) indicate higher concentrations (2667 µM to 297 µM compound concentration), while the darker colours (blue, and violet) indicate lower concentrations (99 to 33 µM). The blue column marks the time range of the cold region and the red one marks the one selected for the hot region.

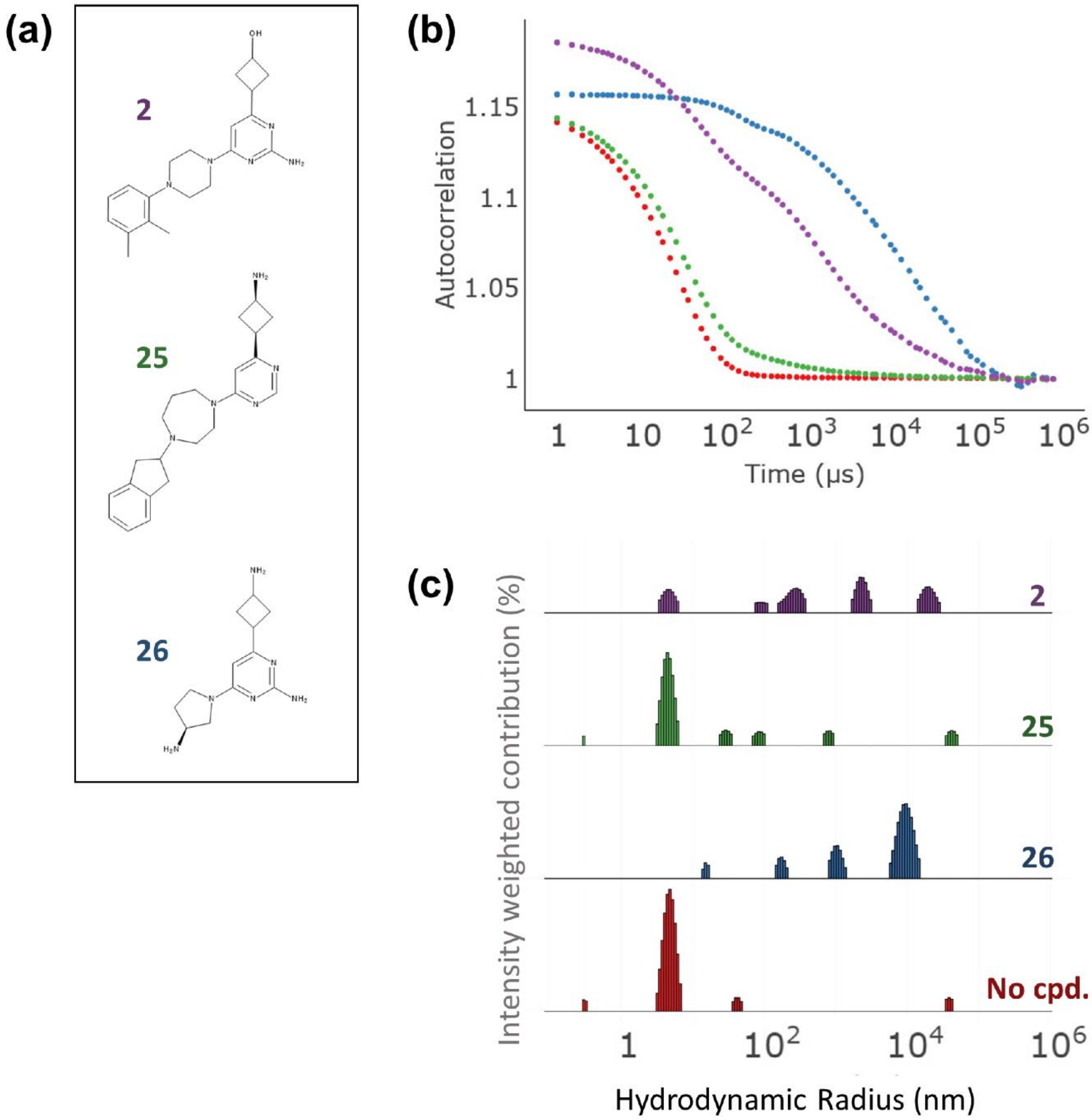

Figure S20. DLS measurements of inactive compounds. (a) Structures of the chosen compounds (b) Mean autocorrelation curves (n=3) measured by DLS of compounds 2 (violet), 25 (green) and 26 (blue) at 890 µM and 0 µM (red). (c) Derived histograms of compounds 2 (violet), 25 (green) and 26 (blue) at 890 µM and 0 µM (red) using the online tool Raynals (https://spc.embl-hamburg.de/).

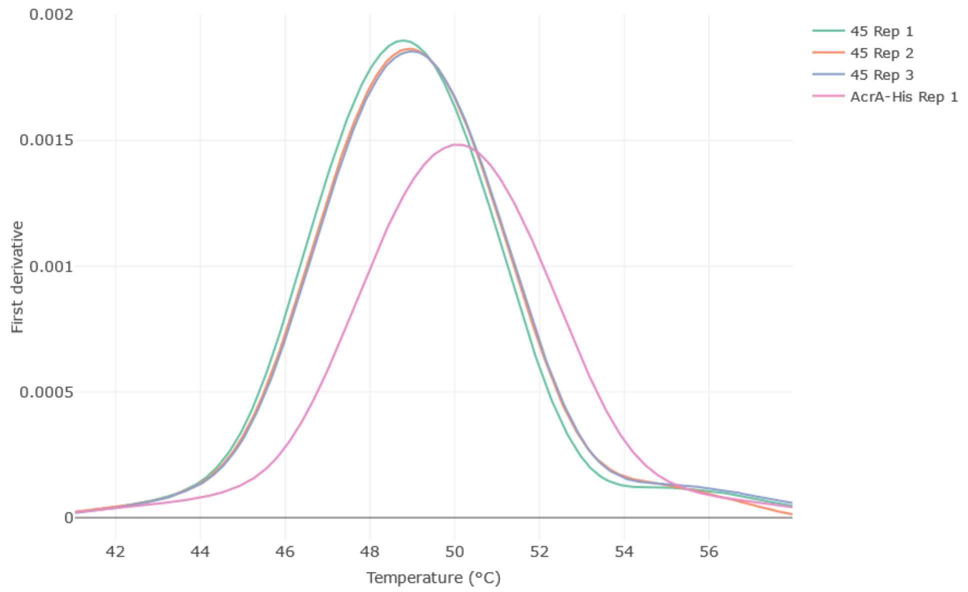

Figure S21. First derivative of the melting curve (nDSF) of 5 µM $AcrA_{His}$ in presence of 2 mM compound 45 (n=3).

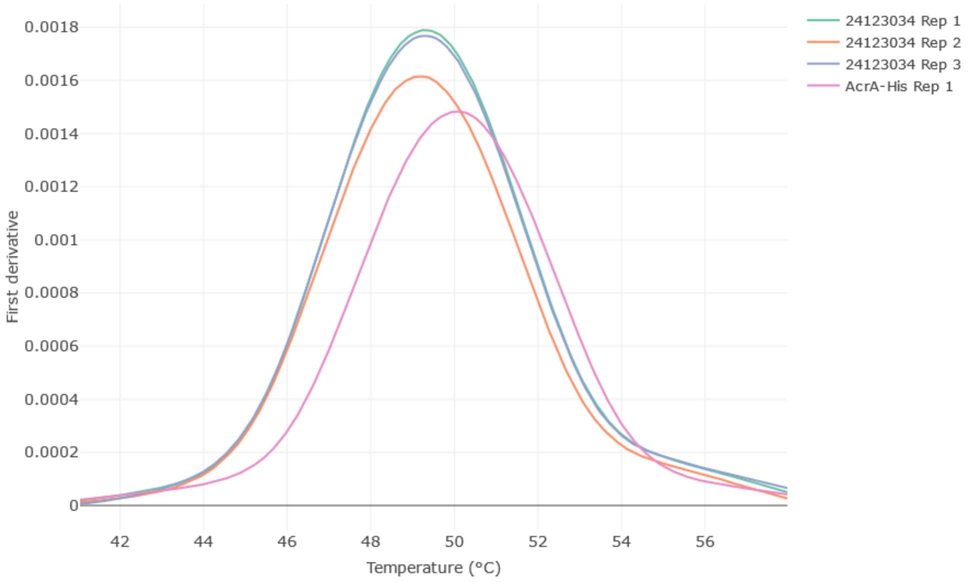

Figure S22. First derivative of the melting curve (nDSF) of 5 µM $AcrA_{His}$ in presence of 2 mM compound 24123034 (n=3).

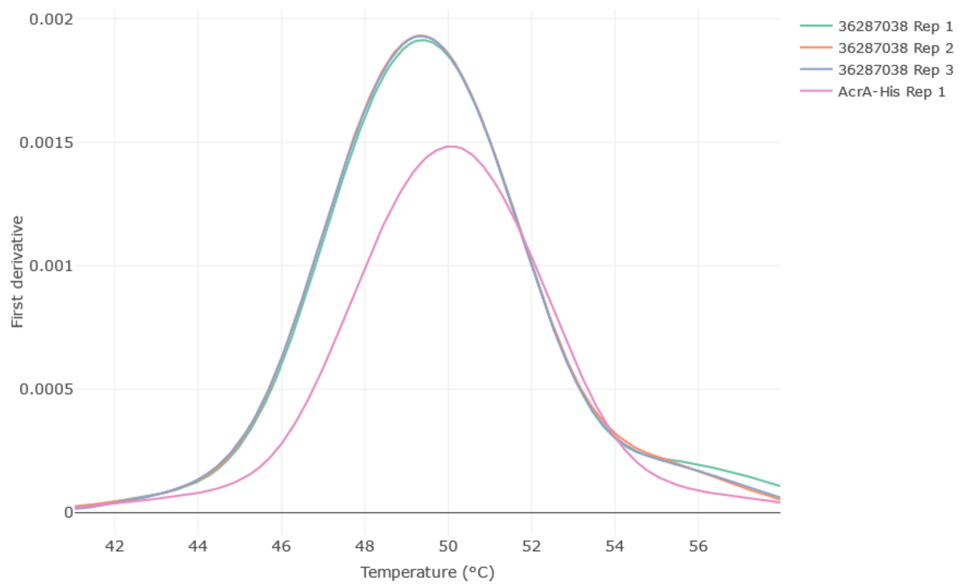

Figure S23. First derivative of the melting curve (nDSF) of 5 µM $AcrA_{His}$ in presence of 2 mM compound 36287038 (n=3).

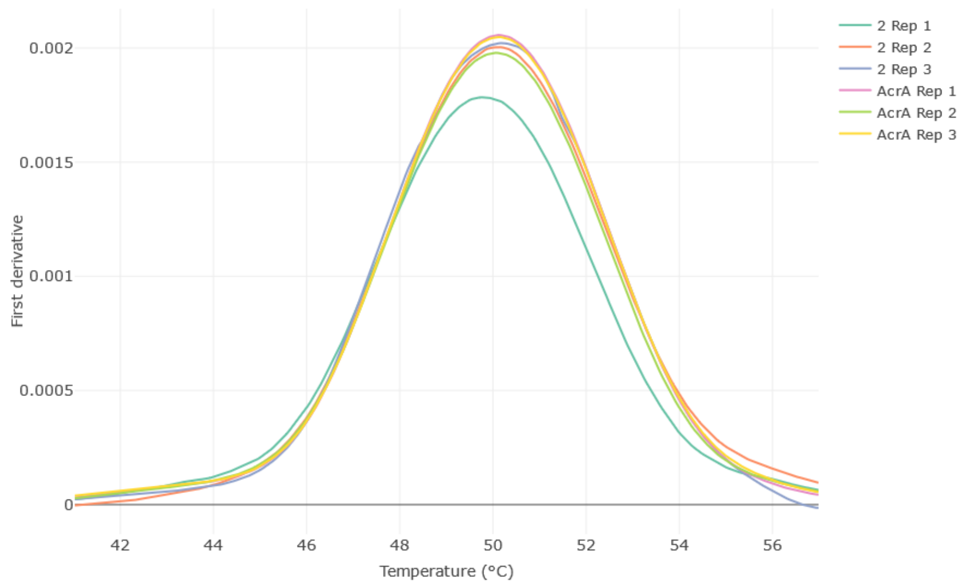

Figure S24. First derivative of the melting curve (nDSF) of 5 µM $AcrA_{His}$ in presence of 1 mM compound 2 (n=3).

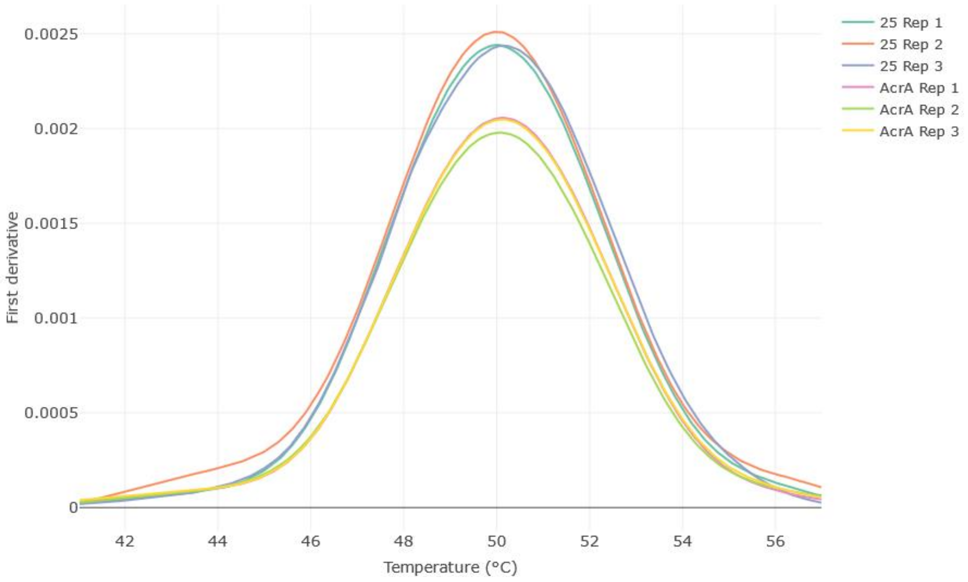

Figure S25. First derivative of the melting curve (nDSF) of 5 µM $AcrA_{His}$ in presence of 2 mM compound 25 (n=3).

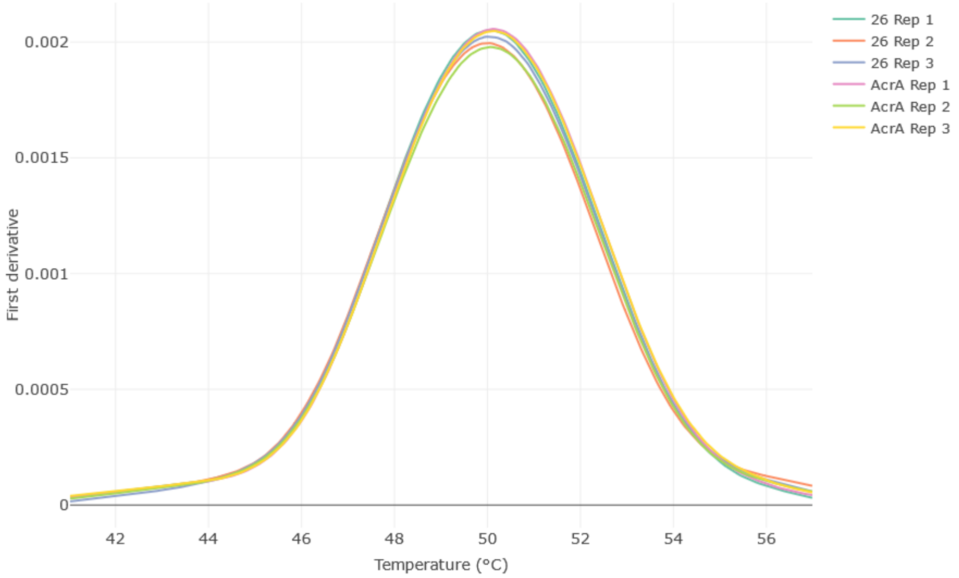

Figure S26. First derivative of the melting curve (nDSF) of 5 µM $AcrA_{His}$ in presence of 2 mM compound 26 (n=3).

Synthesis of compound 24123034

All chemicals, reagents and solvents were purchased from commercial sources and were used without further purification. Thin layer chromatography (TLC) was performed on silica gel using Merck TLC Silca gel 60 F254 Aluminium sheets and was visualized by UV lamp. The direct phase flash column chromatography was carried out using Kieselgel silica gel (35 -70 microm). The reverse phase silica gel chromatography was performed using Biotage purification system with prepacked columns. NMR spectra were recorded on 400 MHz Bruker spectrometer with chemical shift values (δ) in parts per million using the residual solvent signal as an internal standard. The multiplicities are denoted as follows: s, singlet; d, doublet; t, triplet; q, quartet; p, pentet; m, multiplet, br s, broad singlet. HRMS analyses were performed on a hybrid quadrupole time -of -flight mass spectrometer equipped with an electrospray ion source. The purity of all the tested compounds was estimated by HPLC (Empower 3 or SHIMADZU LabSolutions, UV detection at λ = 210 nm) analysis on an Adamas C18 column size: 4.6 x 150 mm, mobile phase: MeCN - 0.1% H_3PO_4, flow rate: 1.0 ml/ min, detector: UV 210 nm and UV 254 nm column temperature: 40°C, sample concentration: 0.5 mg/ml.

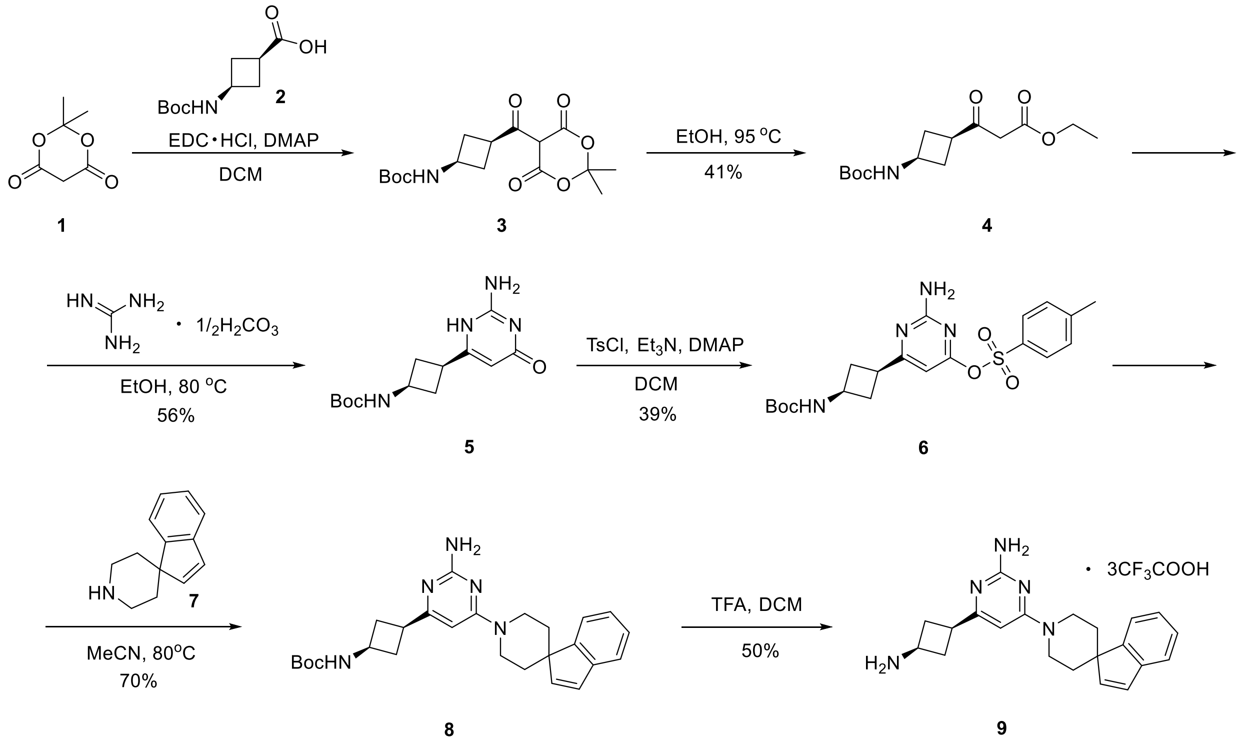

Ethyl 3-(cis-3-((tert-butoxycarbonyl) amino) cyclobutyl)-3-oxopropanoate (4). Meldrum's acid (**1**) (0.48 g; 3.3 mmol) and DMAP (0.55 g; 4.5 mmol) were added to a solution of cis-3-((tert-butoxycarbonyl) amino) cyclobutane-1-carboxylic acid (**2**) (0.65 g; 3.0 mmol) in DCM (15 mL) and cooled in an ice bath before adding EDC·HCl (0.81 g; 4.2 mmol). The reaction mixture was stirred for 24 h at room temperature and diluted with 80 mL DCM. The organic solution was washed with 5% $KHSO_4$ (4x30 mL), H_2O (2x20 mL) to pH ~7 and brine (20 mL), dried over Na_2SO_4, and then concentrated in vacuo. Intermediate **3** was dissolved in EtOH (10 mL), heated for 5 hours at 95 ℃ and concentrated in vacuo. The residue was purified by silica gel chromatography, using DCM:EtOH 100:1.25 / 100:1.5 (v/v) as a mobile phase following by reverse phase chromatography MeCN in H_2O as a mobile phase (linear gradient from 0%:100% to 90%:10%) to give product **4** as a yellow oil (0.35 g; 41%). ^{1}H-NMR (400 MHz, $CDCl_3$) δ: 4.79 (s, 1H), 4.21 – 4.02 (m, 3H), 3.38 (s, 2H), 3.01 (p, *J* = 8.8 Hz, 1H), 2.57 – 2.46 (m, 2H), 2.09 – 1.97 (m, 2H), 1.40 (s, 9H), 1.28 – 1.21 (m, 3H) ppm.

Tert-butyl (cis-3-(2-amino-6-oxo-3,6-dihydropyrimidin-4-yl) cyclobutyl) carbamate (5). Compound **4** (0.35 g; 1.2 mmol) and guanidine carbonate (0.24 g; 2.7 mmol) were dissolved in EtOH (1.2 mL) and heated for 24 h at 80 ℃. The reaction mixture was cooled down and precipitate was collected by filtration to afford the title compound as a white solid (0.19 g; 56%). ^{1}H-NMR (400 MHz, DMSO- d_6) δ: 7.07 (d, *J* = 8.0 Hz, 1H), 6.55 (s, 2H), 5.37 (s, 1H), 3.91 – 3.78 (m, 1H), 2.76 – 2.64 (m, 1H), 2.40 – 2.30 (m, 2H), 1.98 – 1.86 (m, 2H), 1.36 (s, 9H) ppm. ^{13}C-NMR (101 MHz, DMSO-d_6) δ: 170.8, 164.0, 156.0, 154.5, 97.8, 77.6, 41.2, 35.3, 33.4, 28.3 ppm.

2-amino-6-(cis-3-((tert-butoxycarbonyl) amino) cyclobutyl) pyrimidin-4-yl 4-methylbenzenesulfonate (6). To a solution of compound **5** (0.96 g; 3.4 mmol) and TsCl (1.31 g; 6.9 mmol) in DCM (30 mL), Et_3N (1.4 mL; 10.3 mmol), and DMAP (0.08 g; 0.7 mmol) were added. The reaction mixture was stirred for 48 h at room temperature and concentrated in vacuo, dissolved in EtOAc (50 mL), washed with H_2O (2x20 mL), brine (20 mL) and dried over Na_2SO_4. The technical product was purified by silica gel chromatography using DCM:EtOH 100:1.5 (v/v) as a mobile phase (0.58 g; 39 %). ^{1}H-NMR (400 MHz, DMSO-d_6) δ: 7.99 – 7.91 (m, 2H), 7.51 – 7.43 (m, 2H), 7.11 (d, *J* = 8.1 Hz, 1H), 7.00 – 6.90 (m, 2H), 6.14 (s, 1H), 3.96 – 3.82 (m, 1H), 3.00 – 2.88 (m, 1H), 2.47 – 2.38 (m, 2H), 2.42 (s, 3H) 2.04 – 1.92 (m, 2H), 1.36 (s, 9H) ppm.

Tert-butyl (cis-3-(2-amino-6-(spiro[indene-1,4'-piperidin]-1'-yl) pyrimidin-4-yl) cyclobutyl) carbamate (8). Compound **6** (0.41 g; 0.95 mmol) was dissolved in MeCN (10 mL) and spiro[indene-1,4'-piperidine] **(7)** (0.32 g; 1.4 mmol) and Et_3N (0.48 mL; 3.4 mmol)

were added to the solution and heated for 24 h at 80 °C. The reaction mixture was cooled down, the precipitate was collected by filtration and dissolved in EtOAc (30 mL). The organic layer was washed with H_2O (2x20 mL), brine (15 mL), dried over Na_2SO_4 and filtered, the solvent was removed under vacuum resulting in product **8** as a white solid (0.30 g; 70%). ^{1}H-NMR (400 MHz, DMSO-d_6) δ: 7.42 (d, *J* = 7.4 Hz, 1H), 7.35 (d, *J* = 7.2 Hz, 1H), 7.26 – 7.20 (m, 1H), 7.19 – 7.07 (m, 3H), 6.86 (d, *J* = 5.7 Hz, 1H), 6.11 – 5.94 (m, 3H), 4.44 (d, *J* = 13.3 Hz, 2H), 3.95 – 3.81 (m, 1H), 3.30 – 3.22 (m, 2H), 2.84 (t, *J* = 10.0, 7.7 Hz, 1H), 2.48 – 2.36 (m, 2H), 2.12 – 1.92 (m, 4H), 1.38 (s, 9H), 1.27 (d, *J* = 13.2 Hz, 2H) ppm.

4-(cis-3-aminocyclobutyl)-6-(spiro[indene-1,4'-piperidin]-1'-yl) pyrimidin-2-amine trifluoracetic acid salt (9). The solution of compound **8** (0.30 g; 0.67 mmol) in DCM (5 mL) was cooled in an ice bath before adding TFA (2.0 ml; 27 mmol). The reaction mixture was stirred for 3 h at room temperature, concentrated in vacuo and purified by reverse phase chromatography using MeCN in 0.1% TFA (linear gradient from 10%:90% to 90%:10%) as a mobile phase to give product **9** as a white solid (230 mg; 50%). ^{1}H-NMR (400 MHz, DMSO-d_6) δ: 7.31 – 7.24 (m, 2H), 7.21 – 7.14 (m, 3H), 5.85 (s, 3H), 4.29 (d, *J* = 13.1 Hz, 2H), 3.21 – 3.12 (m, 3H *signal overlaps with H_2O*), 2.68 (t, *J* = 11.6 Hz, 3H), 2.41 – 2.30 (m, 2H), 1.86 – 1.69 (m, 3H), 1.62 – 1.53 (m, 2H), 1.05 (qd, *J* = 12.4, 4.1 Hz, 2H) ppm. ^{13}C-NMR (101 MHz, DMSO-d_6) δ: 171.3, 163.2, 163.0, 162.7, 140.2, 129.0, 128.2, 125.9, 89.6, 44.3, 43.6, 42.3, 37.8, 33.6, 31.3 ppm. HRMS (m/z): $[M+H]^+$ calculated for $C_{20}H_{28}N_5$: 338.2345. Found: 338.2348.

^{1}H-NMR (400 MHz, $CDCl_3$), compound **4**

OSM3-LP-73-K3-2.10.1.1r

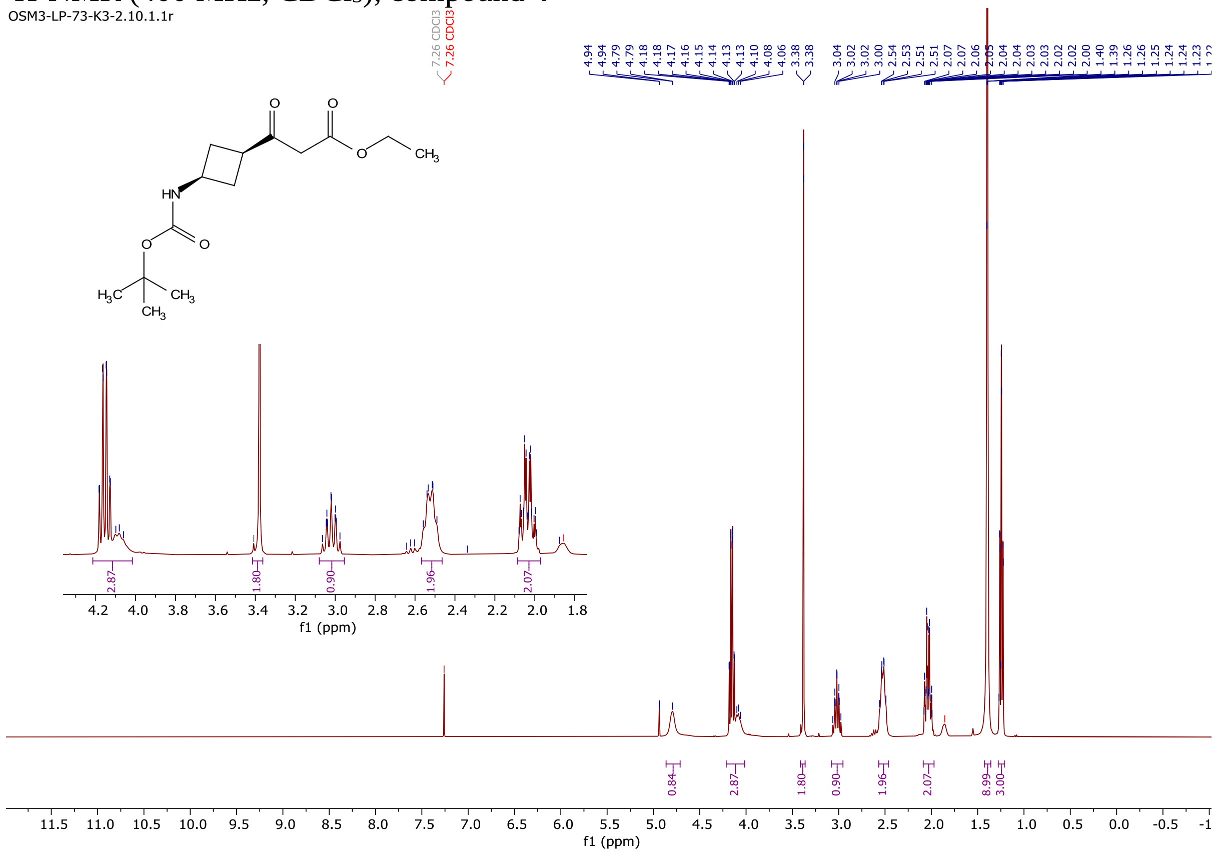

^{1}H-NMR (400 MHz, DMSO-d_6), compound **5**

OSM3-LP-79-6_2021-11-25_PROTON_dmso_01

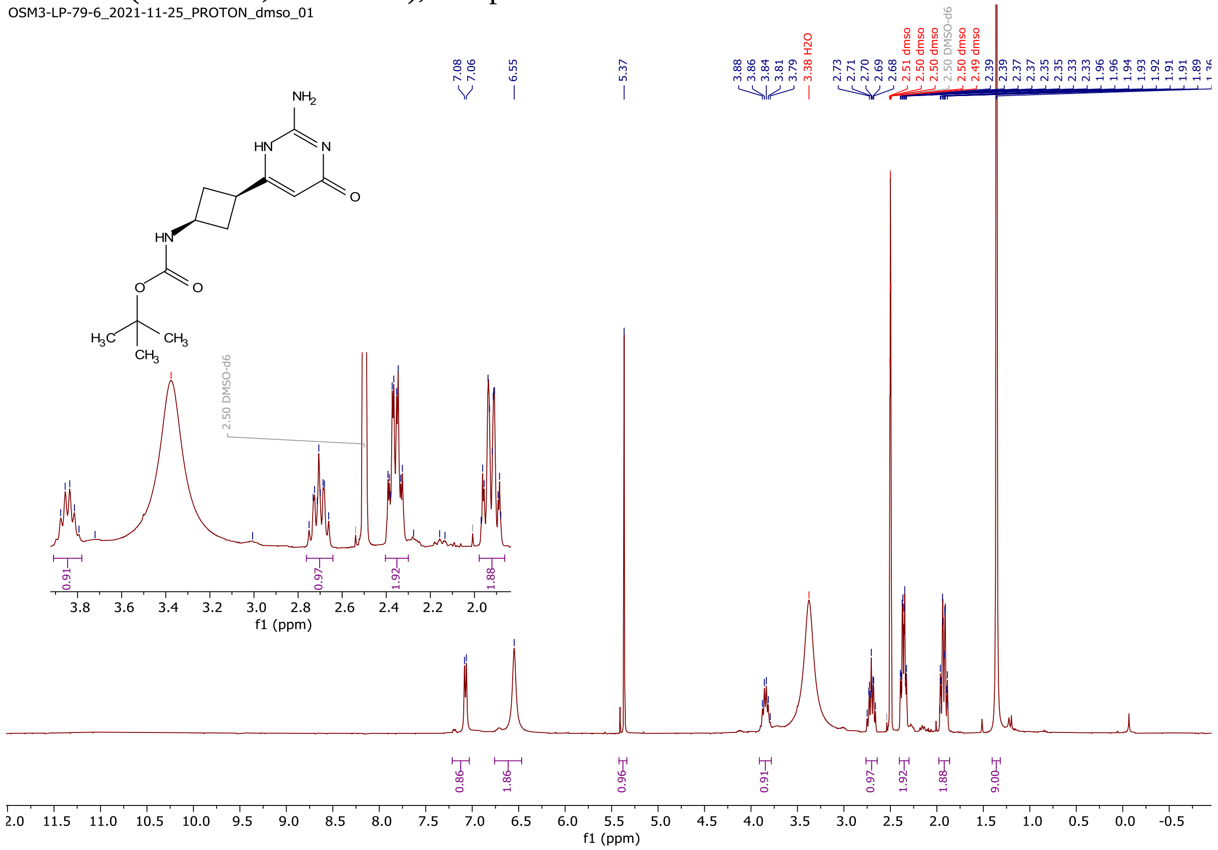

^{13}C-NMR (101 MHz, DMSO-d6), compound **5**

OSM3-LP-79-6-N2.24.1.1r

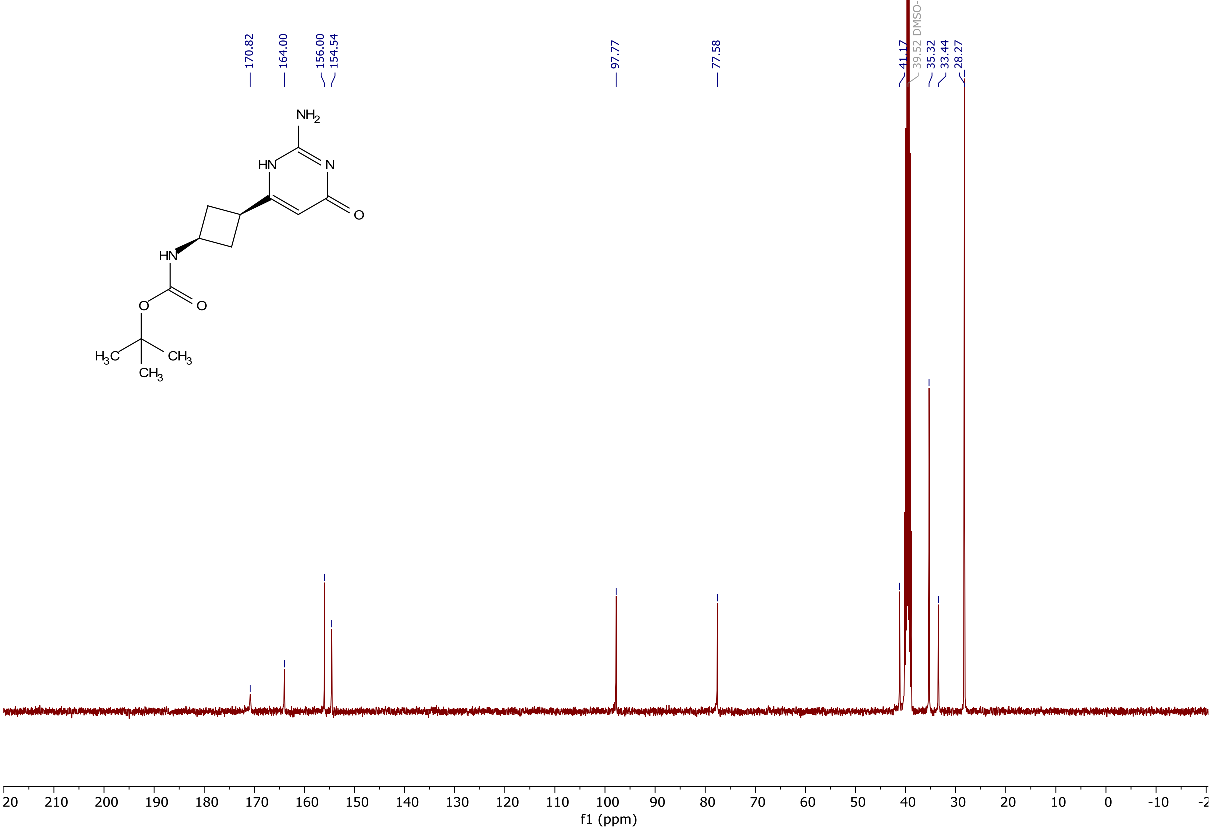

^{1}H-NMR (400 MHz, DMSO-d6), compound **6**

OSM3-LP-94-1.10.1.1r

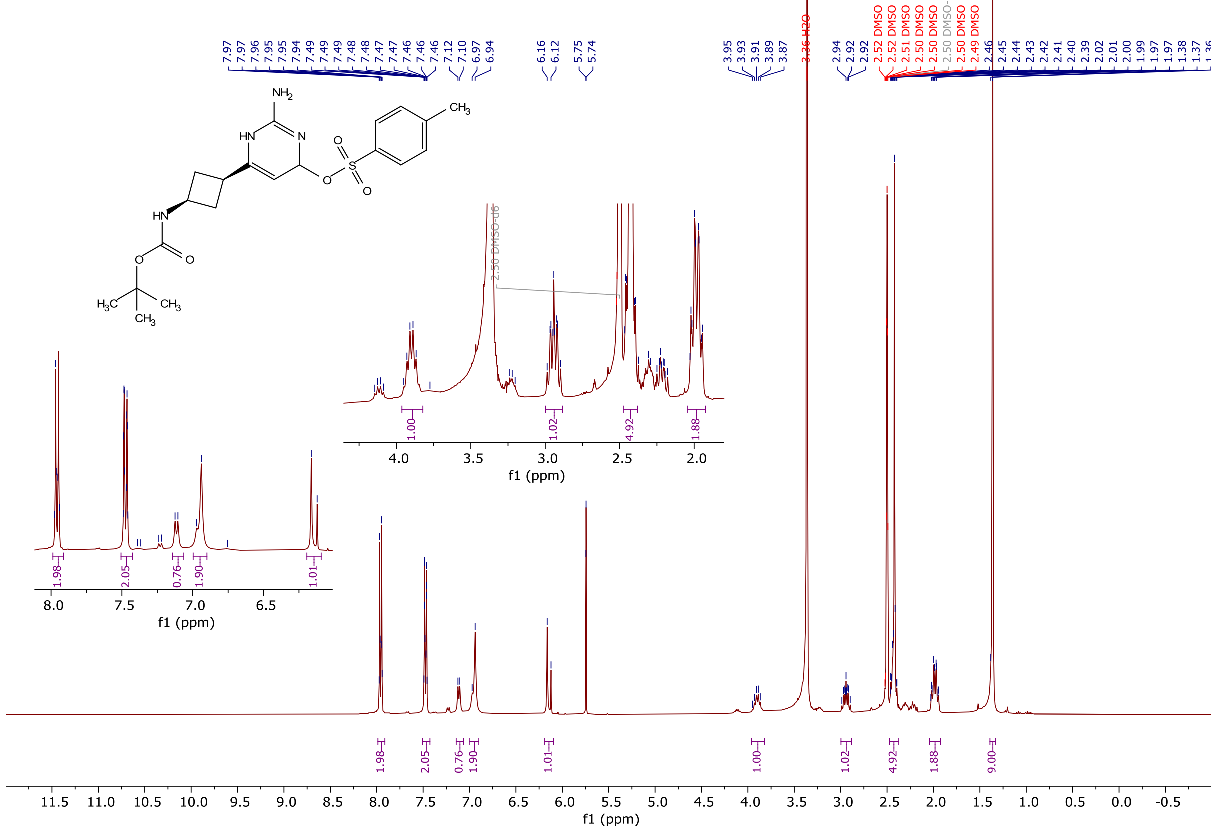

^{1}H-NMR (400 MHz, DMSO-d6), compound **8**

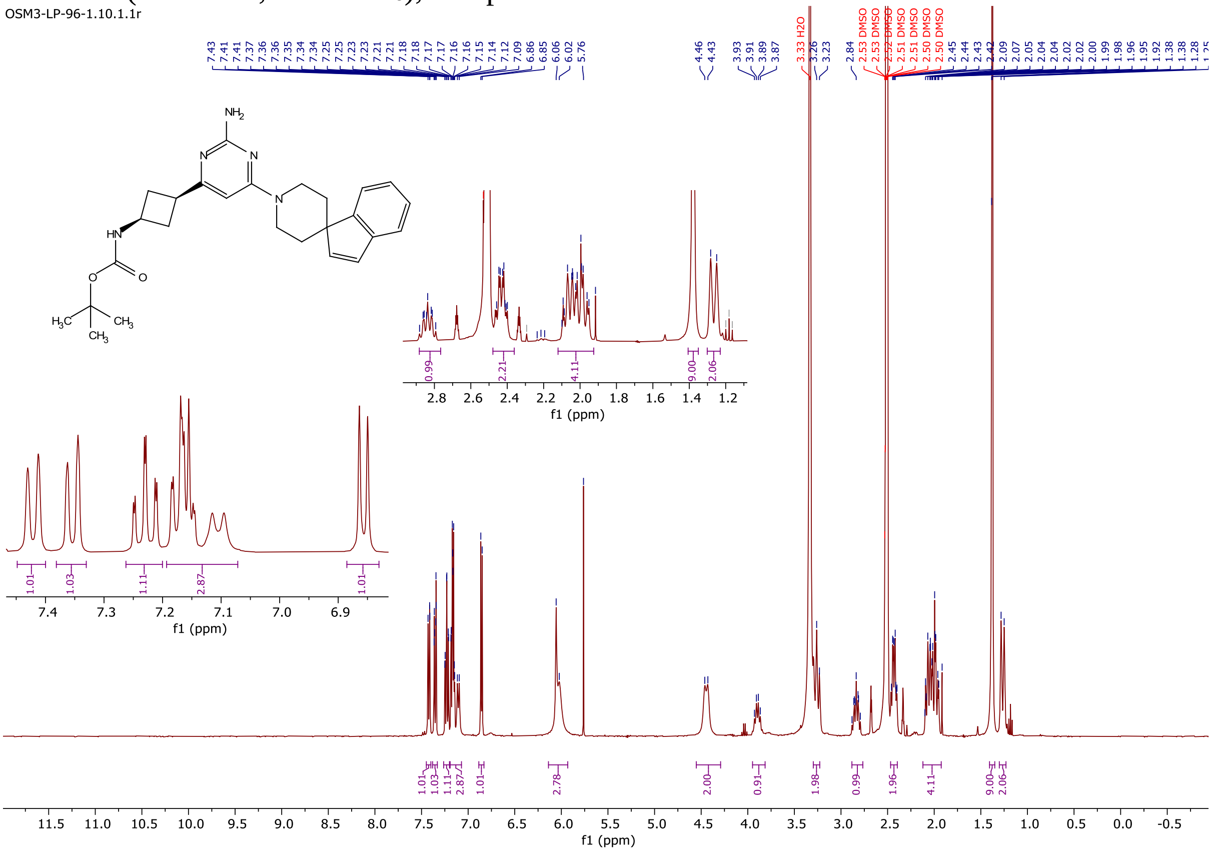

^{1}H-NMR (400 MHz, DMSO-d6), compound **9**

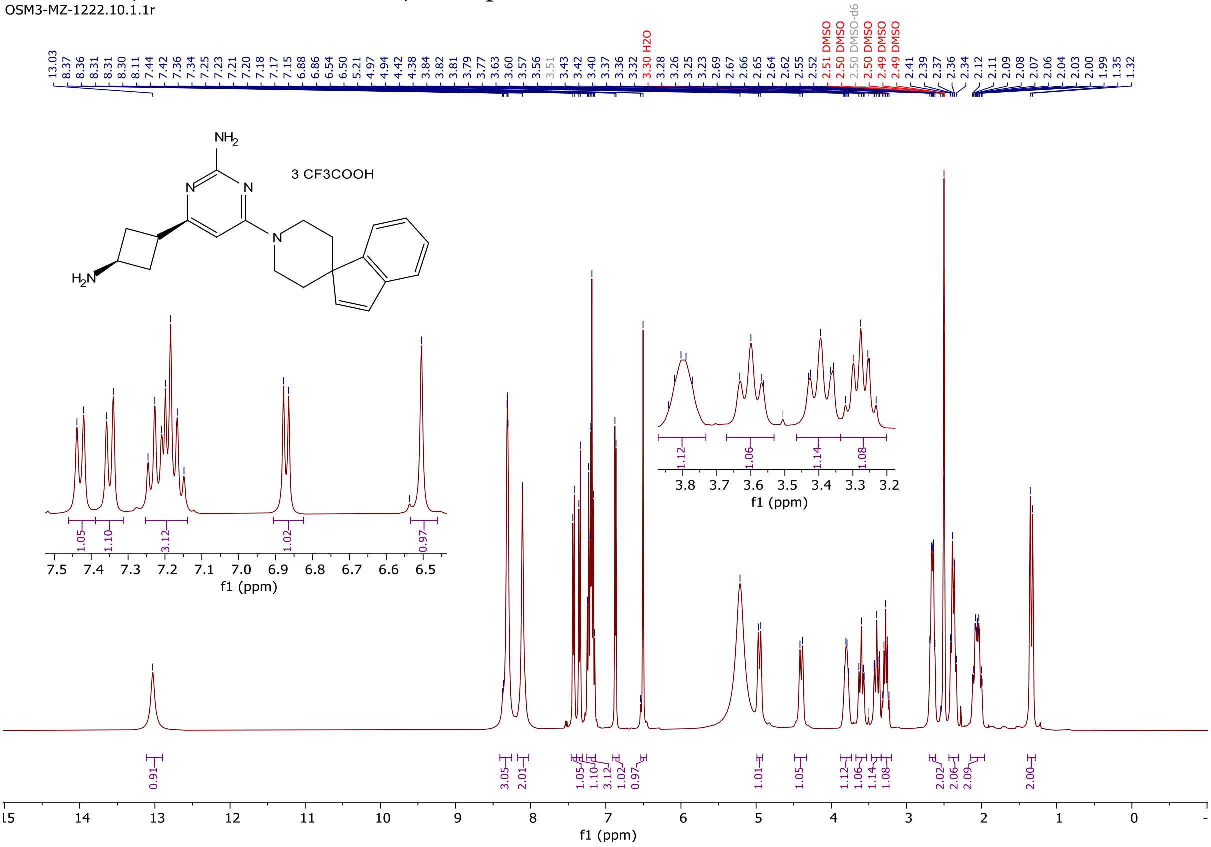

^{13}C-NMR (101 MHz, DMSO-d$_6$), compound **9**

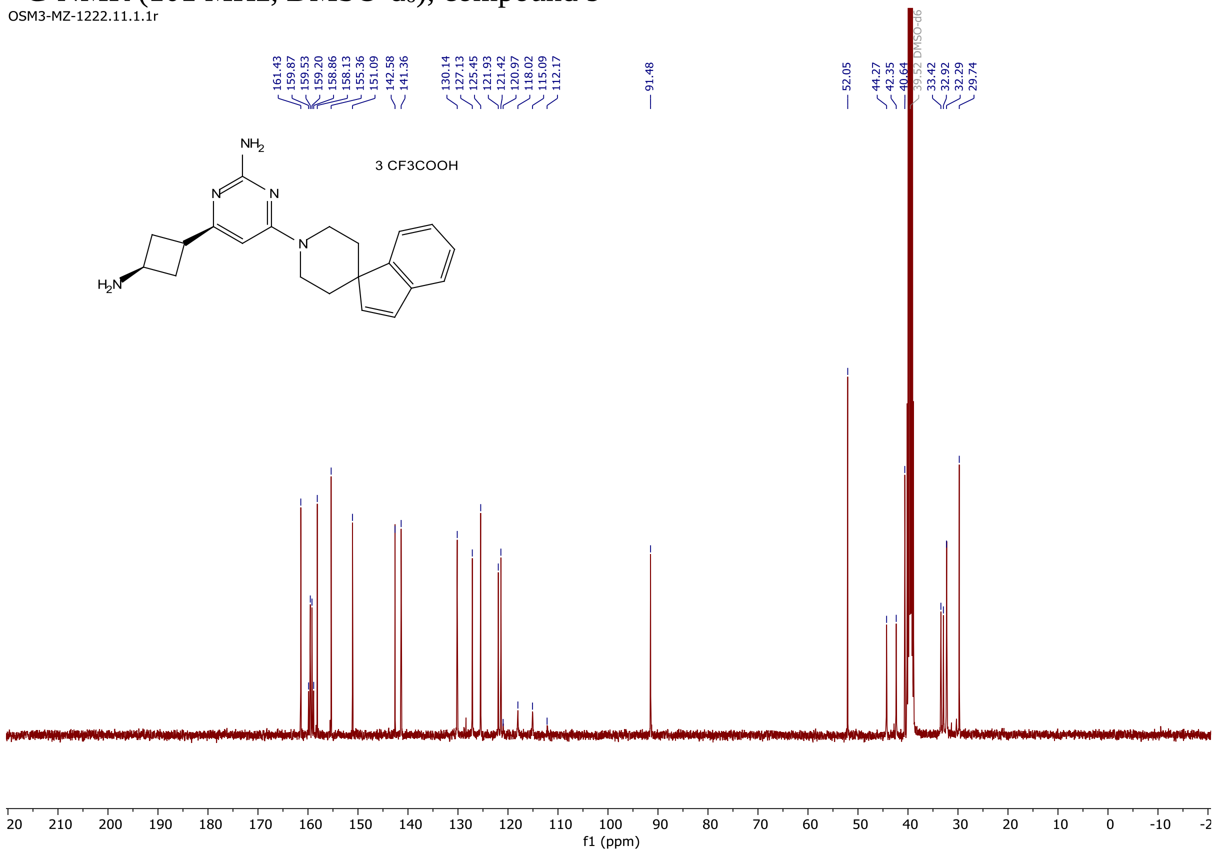

Supplement: Supplementary file 1 [file biomolecules-13-01000-s001.zip › biomolecules-2251755-supplementary.pdf]
